# Use of trihalomethanes as a surrogate for haloacetonitrile exposure introduces misclassification bias

**DOI:** 10.1016/j.wroa.2021.100089

**Published:** 2021-01-22

**Authors:** Kirin E. Furst, Jose Bolorinos, William A. Mitch

**Affiliations:** a Department of Civil and Environmental Engineering, Stanford University, 473 Via Ortega, Stanford, CA, 94305, United States; b Department of Civil, Environmental and Infrastructure Engineering, George Mason University, 4400 University Dr, Fairfax, VA, 22030, United States

**Keywords:** Disinfection byproducts, Drinking water, Epidemiology, Exposure assessment methods, Drinking water monitoring data

## Abstract

Epidemiologists have used trihalomethanes (THMs) as a surrogate for overall disinfection byproduct (DBP) exposure based on the assumption that THM concentrations are proportional to concentrations of other DBP classes. Toxicological evidence indicates THMs are less potent toxins than unregulated classes like haloacetonitriles (HANs). If THMs are not proportional to the DBPs driving toxicity, the use of THMs to measure exposure may introduce non-trivial exposure misclassification bias in epidemiologic studies. This study developed statistical models to evaluate the covariance and proportionality of HAN and THM concentrations in a dataset featuring over 9500 measurements from 248 public water systems. THMs only explain ∼30% of the variance in HANs, whether the data is pooled in a classic linear regression or hierarchically grouped by water system in a multilevel linear regression. The 95% prediction interval on HANs for the median THM concentration exceeds the interquartile range of HANs. Mean HAN:THM ratios range from ∼2.4% to ∼80% across water systems, and varied with source water category, season, disinfectant sequence and distribution system location. The HAN:THM ratio was 265% higher in groundwater systems than in surface water systems and declined by ∼40% between finished effluent and maximum residence times in surface water systems with chlorine-chlorine disinfection. A maximum likelihood approach was used to estimate the misclassification bias that may result from using THMs to construct risk-ratios, assuming that HANs represent the “true” DBP exposure risk. The results indicate an odds ratio of ∼2 estimated with THM concentrations could correspond to a true odds ratio of 4–5. These findings demonstrate the need for epidemiologic studies to evaluate exposure by measuring DBPs that are likely to drive toxicity.

## Graphical abstract

**Figure:**
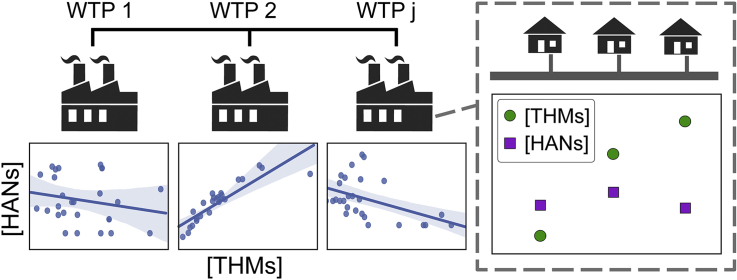

## 1 Introduction

Epidemiologic studies have employed the four chlorinated and brominated trihalomethanes (THMs) as a surrogate for exposure to total disinfection byproducts (DBPs) (Grellier et al., 2015). The significance and magnitude of the association between high THM concentrations and bladder cancer (Costet et al., 2011), colorectal cancer (Rahman et al., 2010), and adverse reproductive outcomes (Grellier et al., 2010; Wright et al., 2017) has been inconsistent, although meta-analyses suggest that the most consistent association is with bladder cancer (Hrudey et al., 2015). DBPs occur in a complex mixture in disinfected drinking waters, with over 700 species identified, and it remains unknown which classes are toxicity drivers (Li and Mitch, 2018; Richardson and Kimura, 2020).

The toxicological evidence suggests that THMs cannot explain the magnitude of risk identified for human health outcomes like bladder cancer (Bull et al., 2012). THMs were substantially less cytotoxic and genotoxic than most unregulated classes using *in vitro* Chinese hamster ovary (CHO) cell assays (Wagner and Plewa, 2017). When concentrations of individual DBPs in drinking waters were weighted by their CHO cytotoxicity LC_50_ values, haloacetonitriles (HANs) consistently comprised the majority of the toxic potency-weighted concentrations, while THMs contributed very little (Chuang et al., 2019; Furst et al., 2019). Recent research has demonstrated that the CHO cytotoxicity of defined mixtures of regulated and unregulated DBPs is additive (Lau et al., 2020), supporting the use of toxic potency-weighted DBP concentrations to compare the contributions of individual DBPs to cytotoxicity.

Despite indications that THMs are not the primary toxicity drivers, THMs continue to be used to assess DBP exposure based on the assumption that THMs correlate with other DBPs. There are several reasons why this assumption may not be valid. First, differences in precursors between source waters could change the tendency to form THMs vs. other DBPs. For example, while humic materials serve as THM precursors in pristine waters (Liang and Singer, 2003), wastewater and algal-impacted waters feature higher dissolved organic nitrogen concentrations serving as precursors for nitrogenous DBPs, including HANs (Westerhoff and Mash, 2002; Shah and Mitch, 2012).

Second, DBP classes feature distinct formation pathways. Differences in disinfection schemes could promote some classes over others (Chuang et al., 2015; Furst et al., 2018). For example, chlorine is more reactive than chloramines regarding halogen transfer to humic materials to form THMs, and to organic nitrogen to form HANs (Hayes-Larsen et al., 2010; Shah and Mitch, 2012). However, HANs can also form via a separate pathway involving chloramine incorporation into aldehydes (Kimura et al., 2013; Vu et al., 2019). Thus, switching from chlorine to chloramines may reduce THMs to a greater extent than HANs.

Third, THMs increase with distribution system residence time as end products, while other DBPs occur as intermediates (Hua et al., 2020). For example, HANs form as intermediates, hydrolyzing to haloacetamides and haloacetic acids (Yu and Reckhow, 2015). Thus, intermediate DBPs could reach steady-state concentrations or decline while THMs continue to increase. Previous studies of the spatiotemporal covariation of DBPs in distribution systems are limited to analysis of THMs with haloacetic acids (e.g., Hinckley et al., 2005; Evans et al., 2013), and qualitative observations of THMs with unregulated classes (Weinberg et al., 2002; Wei et al., 2010).

If THM concentrations are not reliably proportional to concentrations of toxicity drivers, their use to measure DBP exposure could obscure the magnitude and significance of associations between DBPs and adverse health outcomes (i.e., exposure misclassification bias). Previous studies evaluating the relationship between THMs and unregulated classes suffer from the following limitations: 1) low sample count, with few samples from many water systems (Furst et al., 2019; Krasner et al., 1989) and/or samples from few water systems (Wei et al., 2010); 2) analysis conducted on pooled data from multiple water systems and/or time points without accounting for clustering and spatiotemporal dependencies (Wei et al., 2010; Krasner et al., 2016; Furst et al., 2019); and 3) simplified methods for evaluating covariance (e.g., Pearson or Spearman correlation coefficients; Krasner et al., 1989; Wei et al., 2010; Krasner et al., 2016; Furst et al., 2019). The conclusions of these studies were mixed; for HANs and THMs, Krasner et al. (1989) and Wei et al. (2010) found high correlations (r^2^∼0.9), while Furst et al. (2019) found a lower correlation (r^2^∼0.5–0.6).

The goal of this study is to evaluate the assumption that THMs are sufficient exposure surrogates for other DBP classes. HANs are used as a test case for two reasons: 1) previous research suggests HANs are more geno- and cytotoxic than THMs (Wagner and Plewa, 2017); 2) the concentrations of both the four THMs and four HANs (dichloroacetonitrile (DCAN), bromochloroacetonitrile (BCAN), dibromoacetonitrile (DBAN) and trichloroacetonitrile (TCAN)) are available in the U.S. Environmental Protection Agency’s Information Collection Rule (ICR) database (USEPA United States Environmental Protection Agency, 2000). The ICR database encompasses 296 large US public water systems, most consisting of multiple water treatment plant/distribution system pairs (WTPs). Samples were collected quarterly between July 1997 and December 1998 (i.e., up to six sampling events per WTP) from the finished effluent and four distribution system locations, providing over 10,000 sample records. The database is extensively described in McGuire et al. (2002).

This study has 3 objectives:

1) Evaluate the assumption that THM concentrations are proportional to HAN concentrations, between water systems as well as within individual water systems.
2) Examine how the HAN:THM ratio varies with source water type, season, disinfectant sequence, and distribution system residence time.
3) Estimate the odds ratio bias that may be incurred through misclassifying HAN exposure with THMs as a surrogate, and evaluate the implications for detecting associations between DBPs and adverse health outcomes.

The results have important implications for the use of THM concentrations as an exposure surrogate for other DBP classes in epidemiologic studies.

## 2 Materials and methods

### 2.1 Data preparation

The ICR AUX1 database was retrieved from the EPA data repository (USEPA United States Environmental Protection Agency, 2000); details regarding the initial processing and are provided in Text S1. Sample records missing values for any THM or HAN species were excluded. In many records, concentrations of certain species were left-censored, i.e., below the method reporting limits (MRL) of 1.0 μg/L for each THM and 0.5 μg/L for each HAN. Seven WTPs with only left-censored data for all species were excluded. Remaining left-censored concentrations were replaced by half of the MRL; for a record with left-censored concentrations for all four HANs or THMs, the cumulative replacement value was 1.0 μg/L or 2.0 μg/L, respectively. The replacement values for left-censored concentrations could affect the outcome of models (Francis et al., 2009). A sensitivity analysis was conducted by replacing left-censored concentrations with the full MRL value and repeating key analyses; the conclusions were unchanged (Text S2). The final fraction of left-censored records was 15.4% for HANs and 7.6% for THMs.

Records were included for samples collected from finished effluent (FINISH), three intermediate distribution system locations (AVG1, AVG2, and DSE, or “distribution system equivalent,” selected for comparison with simulated distribution system experiments), and the maximum retention time (MAX). Seven WTPs with only finished effluent records were excluded. Systems that blended treated effluents from multiple WTPs were excluded. Nearly 19% of records (n = 1835) had no entry for the primary disinfectant field; 14 records were filled based on WTP process train information. 25 records with erroneous entries were corrected. Less than 1% of records were affected. The secondary (residual) disinfectant field was empty for 1.2% of records (n = 115); these were filled with chlorine or chloramines following evaluation of 1) other records for that WTP, 2) relative concentrations of free and total chlorine, and 3) utility process information. Lastly, records from one of five quarters for one WTP were excluded due to unrealistic TCAN concentrations (∼40 μg/L) at all sampling locations; all TCAN concentrations were below the MRL for the other four quarters, with no observable change in water quality or treatment. The final dataset consisted of 9587 records from 412 WTPs representing 248 public water systems.

### 2.2 Selection of water system features included in the final models

We evaluated the impact of four categorical variables on the HAN:THM ratio: source water type, distribution system location, season, and disinfection sequence (Table S1A). Source water types include surface water, groundwater, mixed source waters where none exceeds 80% of the flow, groundwater under the influence of surface water, and purchased/wholesale waters. Following screening, 71% of records (n = 6772) represented surface water, 25% represented groundwater (n = 2405), and the remaining categories each represented 1–2% of records (Text S1). Records were sorted into season by sampling month: summer (June–August), autumn (September–November), winter (December–February), and spring (March–May). The ICR database included distribution system residence time estimates, but these estimates are imprecise (Text S1). Instead, the location category was used to indicate relative residence time: FINISH < [DSE, AVG1, AVG2] < MAX (Table S1B). Primary disinfectants used were chlorine, chloramines, chlorine-chloramines, chlorine dioxide, ozone, or no entry. Residual disinfectants were either chlorine or chloramines. Primary and residual disinfectants were concatenated to create the disinfectant sequence variable (Table S1A).

### 2.3 Multilevel model development

Prior statistical evaluations of the ICR database were developed by pooling measurements from multiple WTPs with the assumption that data were generated by independent, random sampling (e.g., Obolensky et al., 2008; Francis et al., 2009). However, the ICR data is hierarchically grouped by WTP; model errors are correlated for measurements within each WTP such that pooling the data produces overconfidence in model estimates. In this study, multilevel/hierarchical regression models (MLMs) were used to accommodate the hierarchical data structure and to estimate the variance between and within groups. The MLMs developed have a 2-level hierarchical structure, with level-1 units (9578 sample records) nested within level-2 groups (412 WTPs). The 412 WTPs have a median of 25 records (25th percentile of 20; 75th percentile of 29, range 3–30).

In this study, the classic and multilevel regression models were designated in two ways. For Objective 1, we first regress HAN concentrations on THM concentrations (i.e., [HANs]=β[THMs]+α) to estimate the variance in HAN concentrations explained by THMs. For the remainder of Objective 1 and for Objective 2, we use the ratio of HAN to THM concentrations as the outcome variable (i.e., $\frac{\left[HANs\right]}{\left[THMs\right]}=\alpha$) to directly evaluate whether HAN and THM concentrations are proportional, i.e., maintain a constant ratio. The variance estimates for the multilevel model fit to the HAN:THM ratio are more robust to left-censoring than the equivalent bivariate multilevel model regressing HANs against THMs (Supplemental Text S2).

Regression coefficients for MLMs can either vary by group or are fixed for the population of groups. Equations (1.1), (1.2)) comprise the bivariate MLM used to evaluate the variance in HAN concentrations explained by THMs. Equation (1.1) describes level-1, where outcome variable $y_{ij}$ is the HAN concentration (log-nM) in sample i from WTP j, explanatory variable $x_{ij}$ is the THM concentration (log-nM) in sample i from WTP j, coefficient $\beta_{j}$ is the slope for WTP j, coefficient $\alpha_{j}$ is the intercept, and $e_{ij}$ is the residual error of sample i in WTP j. Equation (1.2) describes level-2 of the model, in which coefficients $\alpha_{j}$ and $\beta_{j}$ consist of a fixed population parameter fit to all WTPs in the dataset ($\alpha_{0}$ and $\beta_{0}$, respectively), and parameters that are fit to each WTP j ($u_{j}$ and $v_{j}$, respectively); $u_{j}$ and $v_{j}$ were assumed to be uncorrelated. Because the HAN and THM concentrations are log-normally distributed, they were log-transformed prior to modeling for precision in the variance estimates.

(1.1) $$Level\;1:\;y_{ij}=\alpha_{j}+\beta_{j}x_{ij}+e_{ij}$$

(1.2) $$Level\;2:\;\alpha_{j}=\alpha_{0}+u_{j},\;\beta_{j}=\beta_{0}+v_{j}$$

Equations (2.1), (2.2)) describe the univariate model (i.e., a model with no explanatory variables), with the intercept allowed to vary by WTP to model the variance in the HAN:THM ratio between and within WTPs. Equation (2.1) describes level-1 of the model, in which outcome variable $y_{ij}$ is defined as $log\left(\frac{HAN\;(nM)}{THM\;(nM)}\right)$ in sample i from WTP j, coefficient $\alpha_{j}$ is the intercept, and $e_{ij}$ is the residual error of sample i in WTP j. Level-2 (Equation (2.2)) shows that $\alpha_{j}$ consists of population parameter $\alpha_{0}$, a fixed value fit to all WTPs, and $u_{j}$, fit to each WTP j.

(2.1) $$Level\;1:\;y_{ij}=\alpha_{j}+e_{ij}$$

(2.2) $$Level\;2:\;\alpha_{j}=\alpha_{0}+u_{j}$$

To evaluate the effect of key water system features on the HAN:THM ratio, Equation (2.1) was modified to include four categorical explanatory variables (Equation (3.1)): source water type (SRC), sample location (EVNT), season (SEAS), and disinfectant sequence (DIS) (coefficients $\beta_{k}$, $\gamma_{l}$, $\delta_{k}$, and $\pi_{n}$, respectively). Intercept $\alpha_{0j}$ is allowed to vary by WTP as in the previous model (Equation (3.2)). Interaction terms are included for source water type with disinfectant sequence and with season (coefficients $\zeta_{km}$ and $\eta_{kn}$, respectively) to capture differences in the effect of season on different source types, and dependencies between the choice of disinfectant and the source water type.

$$y_{ij}=\alpha_{j}+\underset{k}{\sum }\beta_{k}SRC_{k}+\underset{l}{\sum }\gamma_{l}EVNT_{k}+\underset{m}{\sum }\delta_{k}SEAS_{k}+\underset{n}{\sum }\pi_{n}DIS_{n}+$$

(3.1) $$\underset{k}{\sum }\underset{m}{\sum }\zeta_{km}SRC_{k}SEAS_{m}+\underset{k}{\sum }\underset{n}{\sum }\eta_{kn}SRC_{k}DIS_{n}+e_{ij}$$

(3.2) $$\alpha_{j}=\alpha_{0}+u_{j}$$

### 2.4 Estimating odds ratio bias

We used logistic regression to estimate the potential odds ratio bias that may be incurred from misclassifying exposure with THMs as a proxy for more toxic DBPs, with HANs as an illustrative example. Epidemiologic studies often designate a threshold THM concentration as a binary indicator of low or high DBP exposure. For each ICR record, the “proxy” exposure variable was set to one for THM concentrations above 543 nM (the 90th percentile ICR concentration) and zero otherwise. The “true” exposure was set to one for HAN concentrations above the 90th percentile concentration, 62.4 nM, and zero otherwise.

The probability of a given health outcome Y, P(Y), as a function of the binary THM exposure surrogate, Z, is given by:

(4.1) $$P\left(Y\right)=\frac{\text{exp}\left(\alpha +\lambda Z+\sum_{j}\gamma_{j}W_{j}\right)}{1+\text{ exp}\left(\alpha +\lambda Z+\sum_{j}\gamma_{j}W_{j}\right)}$$

Where λ estimates the odds ratios for Y with exposure surrogate Z, and $W_{j}$ is a set of covariates (i.e., source water and distribution system location). For the “true” DBP exposure X and the unbiased odds-ratio estimate $\;\lambda^{\text{∗}}$:

(4.2) $$P\left(Y\right)=\frac{\text{exp}\left(\alpha +\lambda^{\text{∗}}X+\sum_{j}\gamma_{j}W_{j}\right)}{1+\text{ exp}\left(\alpha +\lambda^{\text{∗}}X+\sum_{j}\gamma_{j}W_{j}\right)}$$

Using equations (4.1), (4.2)), we fit the relationship between exposure proxy Z and “true” exposure X to estimate the true odds ratio ($\lambda^{*}$) values corresponding to odds ratio estimates (λ) determined in epidemiologic studies that used THM as the exposure indicator. A maximum likelihood approach was used to estimate the true odds ratios ($\lambda^{*}$). This required several assumptions: 1) that HANs, not THMs, affect health outcomes, 2) that the prevalence of congenital anomalies and bladder cancer in the general population is low, and 3) that exposure misclassification is unrelated to the likelihood of a health outcome or to covariates $W_{j}$, (i.e., it is non-differential). Further details regarding the methods and assumptions are provided in Text S3.

## 3 Results and discussion

### 3.1 THMs as a simple linear predictor of HAN concentrations

By using THMs as a surrogate for overall DBP exposure, epidemiologic studies implicitly assume that THMs are proportional to more toxic DBPs such as HANs. For studies within single water systems, this assumption requires that THMs are highly correlated with HANs, and the intercept (i.e., the HAN concentration projected for a THM concentration of zero) is small such that the HAN:THM ratio does not vary substantially with THM concentration. For studies that pool data from two or more water systems, this assumption further requires a low variance in slopes and intercepts between water systems, such that THM concentrations are consistently representative of HAN concentrations. If these conditions are not met, the use of THMs as a surrogate may cause exposure misclassification resulting in odds ratio bias.

**Fig. 1:**
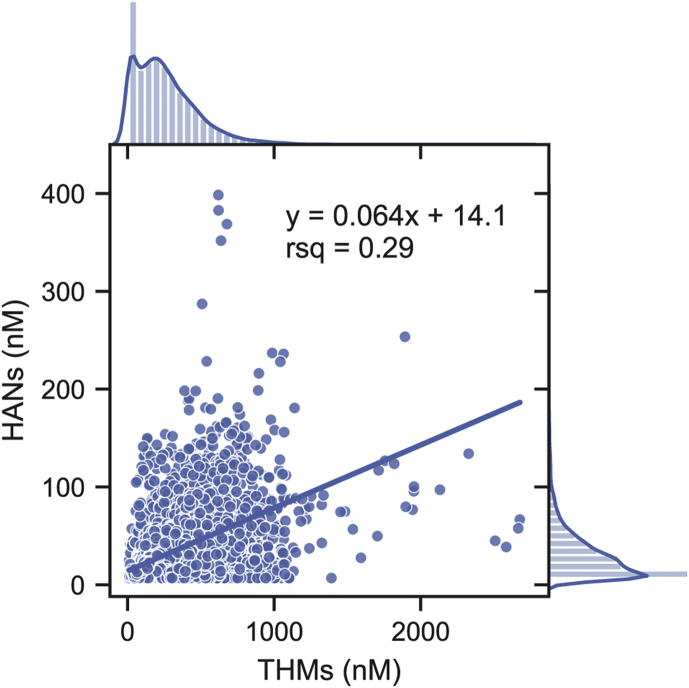
Scatter plot (center) and distributions (margins) of THM and HAN nM concentrations measured in finished effluent and distribution system samples (n = 9587). The OLS line of best fit is y = 0.064x + 14.1 (μg/L: y = 0.066x + 1.8) with Pearson’s correlation coefficient (r^2^) of 0.29 (μg/L: 0.31).

Fig. 1 shows the ordinary least squares (OLS) linear regression of HANs on THMs, where data from 9587 records is pooled without accounting for hierarchical clustering by WTP. The correlation coefficients (r^2^) on a weight (0.31) and molar basis (0.29) indicate that THMs account for only ∼30% of total variation in HANs, substantially lower than the ∼0.9 r^2^-values reported for smaller datasets (Krasner et al., 1989; Wei et al., 2010). For this pooled regression, the error in the predicted HAN concentration for a given THM concentration ($error_{pred}$) is calculated by Equation (5), where $\hat{\sigma }$ is the standard deviation of the residuals, $x_{0}$ is a measured THM concentration, and $var\left(\hat{\beta }\right)$ is the variance of the slope estimate.

(5) $$error_{pred}=\sqrt{{\hat{\sigma }}^{2}+x_{0}^{2}var\left(\hat{\beta }\right)},var\left(\hat{\beta }\right)=\frac{{\hat{\sigma }}^{2}}{\sum_{i=1}^{n}{\left(x_{i}-\bar{x}\right)}^{2}}$$

For the median THM concentration of 31.4 μg/L (223 nM) (Table 1), the predicted HAN concentration is 3.89 μg/L (28.5 nM) with a 95% confidence interval of 0 μg/L to 9.5 μg/L (0 nM–76 nM). For perspective, this interval is 2.6 times the interquartile range of HAN concentrations (3.8 μg/L, 29 nM), indicating that THMs are an imprecise predictor of HANs across multiple water systems.

**Table 1:** Percentiles of THM and HAN concentrations in the ICR dataset.

Statistic	THMs		HANs		HAN:THM ratio	
	μg/L	nM	μg/L	nM	μg/L/μg/L	nM/nM
mean	36.5	265	4.2	31.1	0.182	0.199
standard deviation	29.1	223	3.5	26.7	0.159	0.184
Percentile						
0%∗	2.0	11.6	1.0	6.9	0.00560	0.00490
10%	3.4	19.4	1.0	6.9	0.0476	0.0466
20%	11.6	74.6	1.4	10.1	0.0745	0.0758
25%	15.2	102	1.8	12.6	0.0856	0.0876
30%	18.5	128	2.0	14.6	0.0958	0.0987
40%	25.3	176	2.6	19.2	0.115	0.121
50%	31.4	223	3.3	24.4	0.136	0.143
60%	38.2	276	4.0	29.7	0.158	0.166
70%	46.6	335	5.0	36.7	0.188	0.201
75%	51.1	376	5.6	41.2	0.212	0.227
80%	56.8	418	6.3	46.1	0.244	0.265
90%	74.3	543	8.5	62.4	0.451	0.500
100%	323	2680	52.1	398	1.95	2.17

Table notes: ∗The minimum values are ½ of the MRLs, which replaced left-censored entries for the main analyses in this study.

The fan-shape evident in Fig. 1 suggests that the prediction errors increase with increasing THM concentrations (i.e., the model errors are heteroscedastic), which was confirmed by a Breusch-Pagan test for heteroskedasticity significant at the 0.1% level (Breusch and Pagan, 1979). Heteroscedasticity is often a feature of hierarchically-grouped data where each group has a distinct line of best fit, as would be the case if the HAN:THM relationship varies systematically between water systems.

### 3.2 Variance in the linear relationship of THM and HAN concentrations between water systems

To evaluate the variance between and within the 412 WTPs, Equation (1.1), (1.2) MLM was fit to the ICR dataset with HANs (log-nM) as the outcome and THMs (log-nM) as an explanatory variable to obtain intercept α and slope β for each WTP. The total variance in the regression is decomposed into two components: the level-2 variances, i.e., the systematic variance between the 412 WTP intercepts ($\sigma_{\alpha }^{2}$ = 0.087) and slopes ($\sigma_{\beta }^{2}$ = 0.008), and the level-1 residual variance within WTPs ($\sigma_{e}^{2}$ = 0.128) (Table S2A). The proportion of variance in HANs explained by THMs (R^2^ = 0.322) can be calculated by comparing the total variances in this model with and without THMs as an explanatory variable (Table S3). Thus, even after considering clustering by WTPs, THMs only explain ∼32% of the total variance in HANs.

Across 412 WTPs, the intercepts $\alpha_{j}$ (mean 1.33; 0.295 standard deviation (SD)) and slopes $\beta_{j}$ (mean 0.349; 0.088 SD) obtained from modeling the log-transformed HAN and THM concentrations exhibited substantial variability (Table S2A). The slopes represent the percent increase in HAN concentration for each 1% increase in THM concentration. 95% of the WTP slopes fall between 0.206 and 0.479; thus, for a 1% increase in THMs, the mean increase in HANs is between ∼0.21% and ∼0.48% for most WTPs. To isolate the potential implication of this variability in slopes, we translate to HAN concentrations with the equation $\left[HANs\right]=e^{\alpha }*{\left[THMs\right]}^{\beta }$; for the mean intercept ($e^{1.33}$ = 3.78 nM HANs) and median THM concentration (223 nM), the mean HAN concentration is between 11.5 and 50.7 nM for 95% of WTPs. This interval spans the 20th and 85th percentile HAN concentrations, and easily exceeds the interquartile range (28.6 nM). Therefore, the high variability in slopes relating HAN and THM concentrations between water systems has practical implications for the use of THMs as an exposure surrogate for HANs. The relative standard deviation of the intercepts (0.221) is almost as great as for the slopes (0.252), indicating that variation in baseline HAN concentrations between WTPs may be substantial enough to violate the assumption of proportionality between THMs and HANs.

### 3.3 Evaluation of the assumption of proportionality of HANs and THMs between and within WTPs

The use of THMs as a proxy for total DBP exposure assumes that concentrations of THMs and other DBPs are proportional, i.e., that they maintain a constant ratio as concentrations increase or decrease. Section 3.2 demonstrated that for many WTPs, the intercepts representing baseline HAN concentrations unassociated with THM formation can be substantial, which suggests that the assumption of proportionality could be violated within water systems. However, the simultaneous variation in both slopes and intercepts among WTPs makes the proportionality of THMs and HANs difficult to evaluate. To test whether the assumption of proportionality results in inaccurate estimations of exposure to HANs, Equation (2.1), (2.2) MLM was fit with the ratio of log-transformed HAN and THM concentrations as the outcome variable.

The mean of the 412 WTP log-HAN:THM ratios is −1.98 with a between-WTP variance (i.e., the systematic variance, $\sigma_{\alpha }^{2}$) of 0.480 and within-WTP variance (i.e., residual variance, $\sigma_{e}^{2}$) of 0.249 (Table S4A). The portion of the total variance attributable to systematic variance is computed by the Intraclass Correlation (ICC), ρ (Equation (6)), which ranges from 0 (no systematic variance) to 1 (all variance is systematic).

(6) $$\rho =\frac{\sigma_{\alpha }^{2}}{\sigma_{\alpha }^{2}+\sigma_{e}^{2}}$$

The ICC for the Equation (2.1), (2.2) MLM is 0.66, indicating that epidemiologic studies spanning multiple WTPs while employing the assumption of proportionality between THMs and unregulated DBPs like HANs may be particularly vulnerable to exposure misclassification bias.

**Fig. 2:**
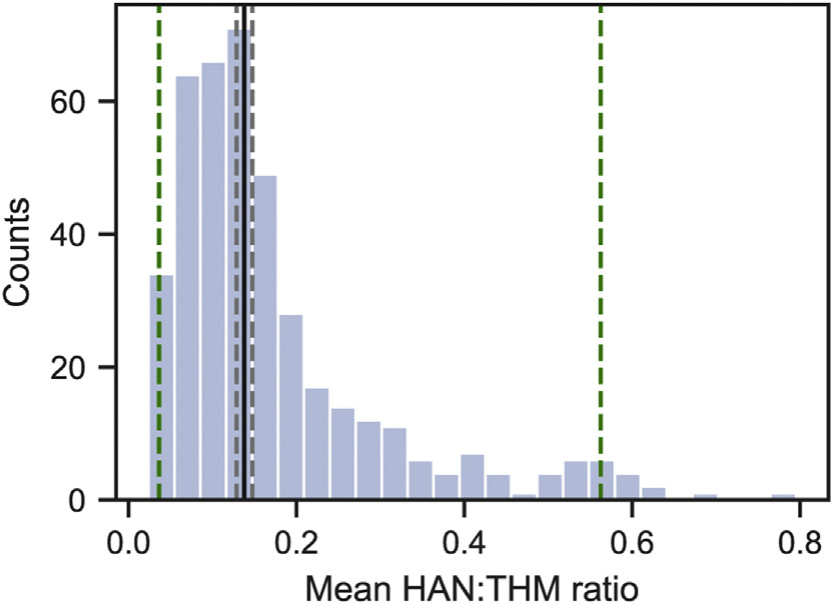
Histogram of geometric mean HAN:THM ratios estimated for 412 WTPs using the multilevel regression model described by Equation (2.1), (2.2). The black solid line indicates the mean, with grey dashed lines indicating the 95% confidence interval for the mean. The green dashed lines indicate the 2.5 and 97.5 percentiles. (For interpretation of the references to color in this figure legend, the reader is referred to the Web version of this article.)

The geometric mean HAN:THM ratio for the population of WTPs is 0.138, while the 412 WTP geometric mean HAN:THM ratios range from 0.0239 to 0.795 (Fig. 2). Thus, depending on WTP, typical HAN concentrations could be anywhere between ∼2.4% and ∼80% of THM concentrations. Even if we exclude outliers by only considering ratios within the 2.5th and 97.5th percentiles [0.0364, 0.563], the median THM concentration (223 nM) corresponds to mean HAN concentrations between 7.7 and 123 nM, an interval four-fold wider than the interquartile range (28.6 nM HANs). Thus, THMs are not proportional to HANs across ICR WTPs, and the variance in the HAN:THM ratio is sufficient to introduce substantial misclassification bias in epidemiologic studies that span multiple water systems.

Considering only within-WTP variance in the HAN:THM ratio (∼34% of the total variance), the HAN:THM ratio ranges from 0.051 (2.5th percentile) to 0.374 (97.5th percentile). For the median THM concentration (223 nM), this translates to a range of 11.3–83.6 nM HANs, which is 2.5-fold larger than the interquartile range of HAN concentrations. Thus, there is substantial variance in the HAN:THM ratio within many water systems, and the assumption that THMs are proportional to HAN concentrations may introduce non-trivial misclassification bias in epidemiologic studies, even when the assumption is employed within one water system.

### 3.4 Evaluation of the contribution of water system features to systematic variance in the HAN:THM ratio

To evaluate whether key water system features can explain the between-WTP and within-WTP variance observed in the HAN:THM ratio, Equation (2.1), (2.2) was adapted to include the following explanatory variables: source water type, season, disinfectant sequence, distribution system location, and the interactions of source water type with season and disinfectant sequence (Equation (3.1), (3.2)). The variances and coefficients estimated by this model are presented in Table S5 and Fig. S1. To evaluate the portion of variance explained by water system features, we compare these variances to those estimated with the univariate model (Section 3.3). The between-WTP variance ($u_{j}$), 0.283, is lower than the univariate model (0.480) by 41%, indicating that these water system features explain a substantial portion of the systematic variance. The within-WTP variance ($e_{ij}$), 0.212, is reduced compared to the univariate model (0.249) by ∼15%. The total variance in the HAN:THM ratio was decomposed into the variance explained by each variable and the residual variance with ANOVA (Analysis of Variance). The water system features and interaction terms each explain a statistically significant share of the total variance in HAN:THM ratios (Table S6).

The mean HAN:THM ratio $\alpha_{0}$ is −2.133 in log-nM units; exponentiating, the conditional geometric mean ratio is 0.119 (nM/nM). Thus under “base-case conditions”, i.e., in finished effluent of WTPs utilizing surface water with chlorine-chlorine disinfection in summer, HAN concentrations are ∼12% of THM concentrations.

#### 3.4.1 Source water

**Fig. 3:**
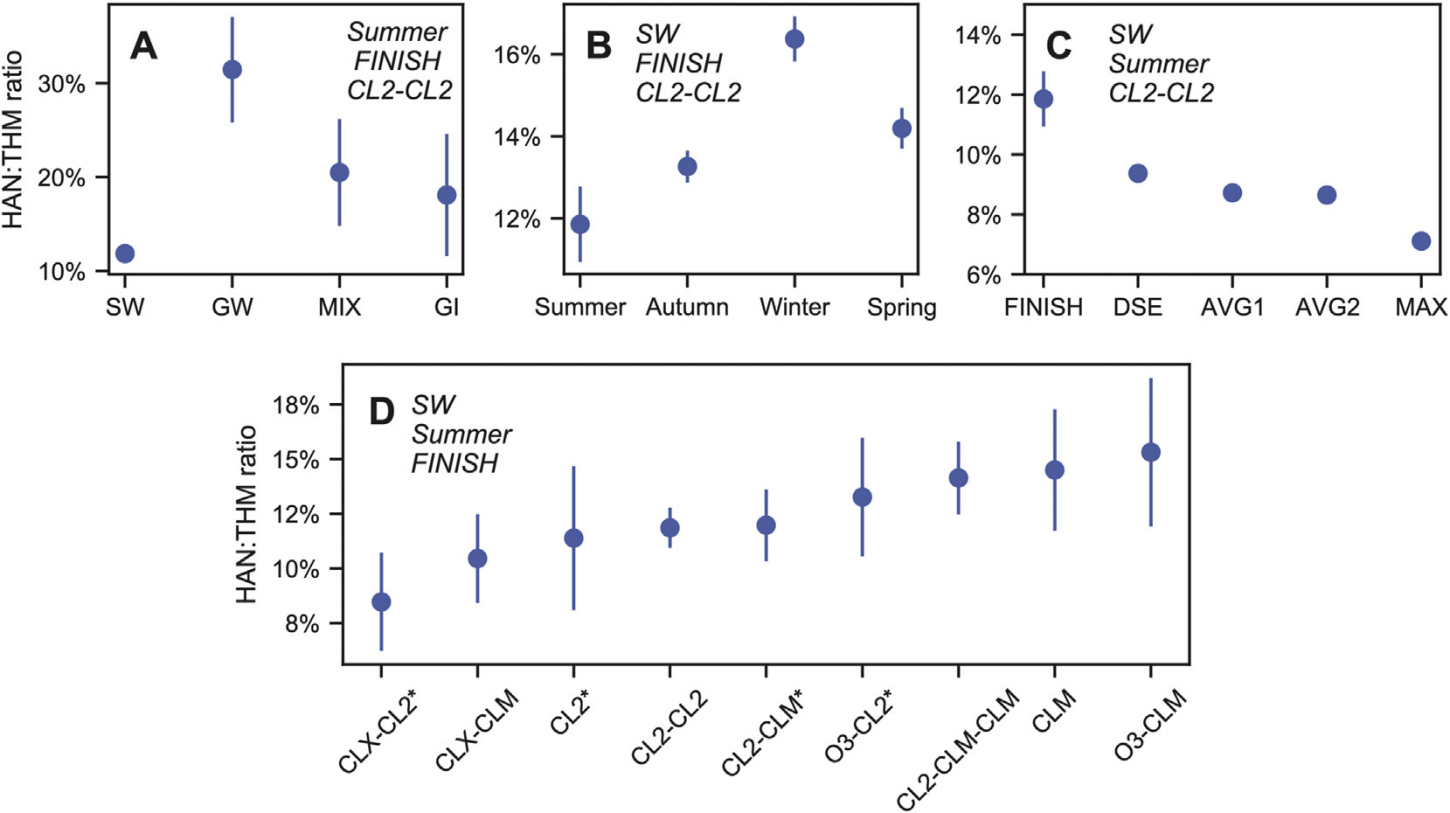
Point plots showing estimated geometric mean HAN:THM ratios for each category of A) source water, B) season, C) disinfectant sequence, and D) distribution system location. Error bars represent 95% CI; all plotted effects were significant (p-value<0.05 or less) with the exception of four disinfectant sequences (Table 2).

Compared to the base-case of surface water, mean HAN:THM ratios for all source water categories were significantly different (p < 0.05), with the exception of purchased/wholesale (Table 2, Fig. 3A). Relative to surface water (0.119), the mean HAN:THM ratio for groundwater (0.315) represents a 165% mean increase in HANs relative to THMs (in finished waters treated with chlorine-chlorine disinfection in the summer). On a molar basis, HAN concentrations are ∼12% of THM concentrations in surface water, but ∼32% of THM concentrations in groundwater. Estimated mean HAN:THM ratios for mixed source water and groundwater under the influence of surface water represent a 73% and 53% increase over surface water, respectively, consistent with their intermediate status as blends of surface water and groundwater. To evaluate whether these effects are primarily driven by differences in HANs or THMs, Equation (3.1), (3.2) MLM was fit with either HAN or THM concentrations (log-nM) as the outcome variable (Fig. S2; Table S7; Table S8). For both HANs and THMs, mean concentrations were lower in groundwater compared to surface water. However, HANs were only 46% lower in groundwater (15 nM) compared to surface water (28 nM) while THMs were 79% lower in groundwater (50 nM) than surface water (237 nM), on average. Thus, the discrepancy in HAN:THM ratios between different source water types is driven by greater differences in THMs relative to HANs. The reasons for the different behavior of HANs and THMs with respect to source water categories is beyond the scope of this model, and warrants further investigation.

**Table 2:** Effects of categorical variables as percent increase or decrease of the mean HAN:THM ratio estimated for surface water.

Category	% Effect (2.5%, 97.5% CI)	Adjusted mean ratio	Sig.
Base-case: SW, FINISH, Summer, CL2-CL2		0.119	∗∗∗
Source water (FINISH, Summer, CL2-CL2)			
GW	165 (122, 217)	0.315	∗∗∗
MIX	72.9 (31.4, 128)	0.205	∗∗∗
GI	52.7 (7.5, 117)	0.181	∗
PUR	24.1 (−30.6, 122)		
Sampling location (SW, Summer, CL2-CL2)			
DSE	−20.9 (−23.2, −18.6)	0.094	∗∗∗
AVG1	−26.4 (−28.5, −24.2)	0.087	∗∗∗
AVG2	−27.0 (−29.1, −24.9)	0.086	∗∗∗
MAX	−40.0 (−41.7, −38.2)	0.071	∗∗∗
Season (SW, FINISH, CL2-CL2)			
Autumn	11.9 (8.7, 15.2)	0.133	∗∗∗
Winter	38.1 (33.6, 42.8)	0.164	∗∗∗
Spring	19.7 (15.7, 24.0)	0.142	∗∗∗
Disinfectant sequence (SW, FINISH, Summer)			
CL2-CLM	1.0 (−11.8, 15.7)		
Cl2-CLM-CLM	19.3 (61, 24.1)	0.141	∗∗∗
CLM	22.3 (1.1, 48.0)	0.145	∗
CLX-CL2	−28.5 (−45.0, −7.2)	0.085	∗
CLX-CLM	−11.8 (−27.3, 6.9)		
CL2	−4.0 (−27.8, 27.7)		
O3-CL2	11.9 (−8.6, 37.0)		
O3-CLM	29.2 (3.7, 61.0)	0.153	∗

Table key: Sig.: significance to the ∗ 95%, ∗∗ 99%, or ∗∗∗ >99.9% confidence level*.* Disinfectant sequence abbreviations: chlorine (CL2), chloramines (CLM), chlorine dioxide (CLX), ozone (O3).

Table notes: Effect estimates in Table S5A were converted to percent effect by exponentiating, subtracting one, and multiplying by 100. To calculate the adjusted mean ratio for a particular category, add 100% to the percent effect and multiply by the conditional mean ratio (i.e., the intercept, the base-case, 0.119).

#### 3.4.2 Seasonal impacts

Statistically significant variation was observed in the HAN:THM ratio across all seasons for the base-case of finished water disinfected with chlorine-chlorine associated with surface water (Table 2, Fig. 3B). The geometric mean HAN:THM ratio is lowest in summer (0.12) and peaks in winter (0.16), a 38% increase. The interaction effects between season and source water type indicate that the HAN:THM ratio does not seasonally vary in groundwater (Table 3). Seasonal variation of THMs in surface waters has been demonstrated, accompanied by the assumption that the changes are representative of the overall DBP mixture (Symanski et al., 2004; Rodriguez et al., 2004). To determine whether seasonal effects on the HAN:THM ratio are due to changes in THMs or HANs, Equation (3.1), (3.2) was refit with either THMs or HANs as the outcome variable. A significant (p < 0.001) reduction in THMs was observed in winter by ∼35% relative to summer for finished surface waters disinfected with chlorine-chlorine (Table S8), while HANs were only ∼10% lower in winter (p < 0.001) (Table S7). Additional research is needed to isolate the causes responsible for the observed seasonal differences in HAN and THM formation.

**Table 3:** Effects of categorical variables as percent increase or decrease of the mean HAN:THM ratio; interactions of groundwater with season and disinfectant sequence.

Explanatory variables	Percent effect (2.5%, 97.5% CI)	Adjusted mean ratio	Sig.
GW, FINISH, Summer, CL2-CL2	165 (122, 217)	0.315	∗∗∗
Season (GW, FINISH, CL2-CL2)			
Autumn	−11.0 (−15.8, −5.8)	0.313	∗∗∗
Winter	−26.6 (−31.3, −21.7)	0.319	∗∗∗
Spring	−14.3 (−19.9, −8.4)	0.323	∗∗∗
Disinfectant sequence (GW, FINISH, Summer)			
CL2-CLM	21.0 (−58.8, 255)		
Cl2-CLM-CLM	−27.8 (−46.3, −3.0)	0.271	∗
CLM	−22.0 (−45.0, 10.6)		
CL2	39.5 (−1.4, 97.4)	0.421	.
O3-CLM	−34.5 (−78.0, 94.7)		

Table key: Sig.: significance to the ∗95% confidence level, ∗∗99% confidence level, and ∗∗∗>99.9% confidence level (”.” indicates near significance at the 95% confidence level).

Table notes: Values for adjusted mean ratios are only provided if the effect was significant. Effect estimates from Table S5B are converted to percentages by exponentiating, subtracting one, and multiplying by 100. To calculate the adjusted mean ratio for a category with groundwater, refer to Table 2 for the adjusted mean ratio of that category with surface water, multiply by 265% (the percent effect for groundwater plus 100%), and finally multiply by the percent effect of that category with groundwater plus 100%.

#### 3.4.3 Disinfectant sequence

The choice of disinfectant sequence is dependent on the source water type, quality, and treatment train (Obolensky et al., 2007). Of 412 WTPs, 146 (35%) used chloramines for secondary and/or primary disinfection during the study period. Some WTPs used chlorine dioxide (21, 5.1%) and ozone (18, 4.4%) for primary disinfection. The ANOVA results show that overall, the portion of the variance due to disinfectant sequence type is statistically significant yet small relative to other predictors (Table S6). Of eight disinfectant sequences used by surface water WTPs, four exhibit statistically significant differences in mean HAN:THM ratios compared to chlorine-chlorine WTPs for finished waters in the summer (Table 2, Fig. 3C). WTPs that used chlorine dioxide-chloramines and ozone-chloramines exhibit 28.5% and 29.2% lower HAN:THM ratios than chlorine-chlorine WTPs. WTPs that used chlorine-chloramines-chloramines or chloramines-only exhibit 19.3% and 22.3% higher HAN:THM ratios, respectively. At groundwater WTPs, the effects of most disinfectant sequences were statistically indistinguishable from surface water WTPs. However, groundwater WTPs that used chlorine-chloramines-chloramines had a 14% lower adjusted mean HAN:THM ratio (0.271) relative to chlorine-chlorine.

To determine whether these effects correspond to trends in individual THM and HAN concentrations, the model was refit with THM or HAN (log-nM) concentrations as the outcome variable (Tables S7 and S8). For surface water WTPs, mean HANs are ∼30% lower and mean THMs are 20–45% lower for WTPs that used four of five chloramine-based disinfectant sequences (chlorine-chloramines, chloramines, chlorine dioxide-chloramines, and ozone-chloramines) compared to chlorine-chlorine. However, mean HAN and THM concentrations at chlorine-chloramine-chloramine WTPs are not significantly distinct from chlorine-chlorine WTPs. These models compare concentrations between WTPs that used chlorine-chlorine and other disinfectant sequences, which is distinct from comparing concentrations between different disinfectants used within the same water system. WTPs that used a chloramine-based disinfectant sequence may not have had the same THM or HAN concentrations with chlorine as WTPs still using chlorine at the time of ICR data collection, particularly as WTPs with higher THMs are more likely to implement chloramines.

#### 3.4.4 Distribution system location

The ANOVA results show that distribution system location explains a notable share of the variance in the HAN:THM ratio, indicated by the high sum of squares (Table S6). Under base-case conditions (surface water disinfected with chlorine-chlorine in the summer), the geometric mean HAN:THM ratio decreased by an average of 40% between FINISH (0.119) and MAX (0.071) sampling locations, and by ∼20–27% between FINISH and the three intermediate sampling locations (Table 2, Fig. 3D). A decline in the HAN:THM ratio with distance from the WTP is consistent with findings that THMs tend to increase with distribution system retention time (Rodriguez et al., 2004), while HANs may peak early and decline with time due to hydrolysis (Yu and Reckhow, 2015). These trends were confirmed by fitting the model with either HANs or THMs as the outcome variable (Tables S7 and S8). Under base-case conditions, THMs increase by ∼35–45% between FINISH and intermediate sampling locations, and ∼64% between FINISH and MAX sampling locations, on average (Fig. S3A). The average change in HANs is small or negligible, with a 7.4% increase between FINISH and AVG2, and no significant difference (p > 0.05) between FINISH and MAX (Fig. S3B).

#### 3.4.5 Practical importance for epidemiological studies

To understand the implications of the effects of these four water system features on the HAN:THM ratio, consider a hypothetical epidemiologic study which uses THMs as an exposure surrogate for more toxic DBPs like HANs. This study encompasses one surface water and one groundwater system, both using chlorine-chlorine in the summer, with mean HAN:THM ratios corresponding to the model estimates (Table 2). We measure 235 nM THMs in the surface water system and 89 nM THMs in the groundwater system, representing a 62% reduction in the exposure surrogate for the groundwater system customers. However, HAN concentrations are 28 nM for both the surface water (0.119 HAN:THM) and groundwater (0.315 HAN:THM) systems. Epidemiologic studies often bracket THM concentrations into tertiles or quartiles. Say the data distribution in our study is identical to the ICR (Table 1); we therefore classify 89 nM in the first tertile or quartile, and 235 nM in the second tertile or third quartile. We also try classifying exposure with a binary contrast, assigning the low (reference) dose to customers of the groundwater system and the high (treatment) dose to customers of the surface water system. Either way, if HANs are the main driver of the health outcome of interest, our study would likely result in a false negative.

Similarly, consider a study within one water system disinfecting with chlorine-chlorine in the summer. Say we measure 235 nM THMs in the finished effluent (or nearest customer location) and 394 nM THMs at the maximum residence time location. We therefore classify exposure as ∼68% higher at the maximum residence time location, but actual HAN concentrations are 28 nM at both locations (FINISH: 0.119, MAX: 0.071 HAN:THM). Say 235–394 nM THMs represents the range of concentrations measured in the study, and we classify exposure by bracketing THM concentrations into tertiles or quartiles. Thus, we assign the nearest customer to the lowest exposure bracket and the maximum residence time customer to the highest bracket. If HANs are the main driver of the health outcome of interest, our study would likely result in a false negative. Say we conduct another study in this system to consider seasonal contrasts. In finished effluent, we measure 235 nM THMs in summer and 171 nM THMs in winter, representing a 38% reduction, but HAN concentrations were 28 nM in both summer (0.119 HAN:THM) and winter (0.164 HAN:THM). If HANs are the main driver of the targeted health outcome, we would accurately find no effect of season on exposure risk, but we would miss-attribute the reason to an insufficient contrast in THM concentrations.

Finally, consider a study of surface water systems using different disinfectant sequences. We measure 235 nM THMs at a facility using chlorine-chlorine disinfection, and 193 nM THMs at a facility using chloramines, and therefore classify exposure as ∼20% lower for the chloramine facility. But HAN concentrations were 28 nM at both the chlorine-chlorine (0.119 HAN:THM) and chloramines (0.145 HAN:THM) facilities. Although the largest effect identified for a disinfectant sequence, 29% (chlorine-chlorine versus ozone-chloramines), is smaller than the maximum effect for the other three water system features, combinations of categorical effects could compound differences in the HAN:THM ratio. For example, if 223 nM THMs were measured in the finished effluents of a surface water facility using chlorine dioxide-chlorine (0.085 HAN:THM) and a groundwater facility using chlorine-chlorine (0.315 HAN:THM), the HAN concentrations would be 19.0 nM (∼40th percentile) and 70.2 nM (>90th percentile), respectively, representing a 270% increase.

### 3.5 Odds-ratio bias from DBP-exposure misclassification

The final objective of this study is to estimate the odds ratio bias that could result from using THMs as a surrogate of exposure to other DBPs in epidemiologic studies, with HANs as an example. If THMs do not accurately measure exposure to HANs, this could result in misclassification of exposure, which could in turn result in systematic biasing of odds ratio estimates. Table 4 presents a confusion matrix in which high THMs (>90th percentile concentrations) are used as an indicator of high HAN exposure (>90th percentile concentrations). Eighty-four percent of ICR records represent true negatives, with low THMs accurately identifying low HAN exposure. Of the high-HAN observations, only 41% were correctly classified as high-exposure by the THM indicator, while 59% were incorrectly classified as low-HAN exposure (false negatives). Conversely, 59% of high-THM observations correspond to low-HAN exposure (false positives). This demonstrates that using high THMs as an indicator of high HAN exposure can introduce exposure misclassification in the form of both false negatives and false positives.

**Table 4:** Confusion matrix showing the percentage of sampling events (n = 9586) with false negatives and false positives in the ICR dataset.

	THM Exposure = 0	THM Exposure = 1
HAN exposure = 0	84%	5.9%
HAN exposure = 1	5.9%	4.1%

**Fig. 4:**
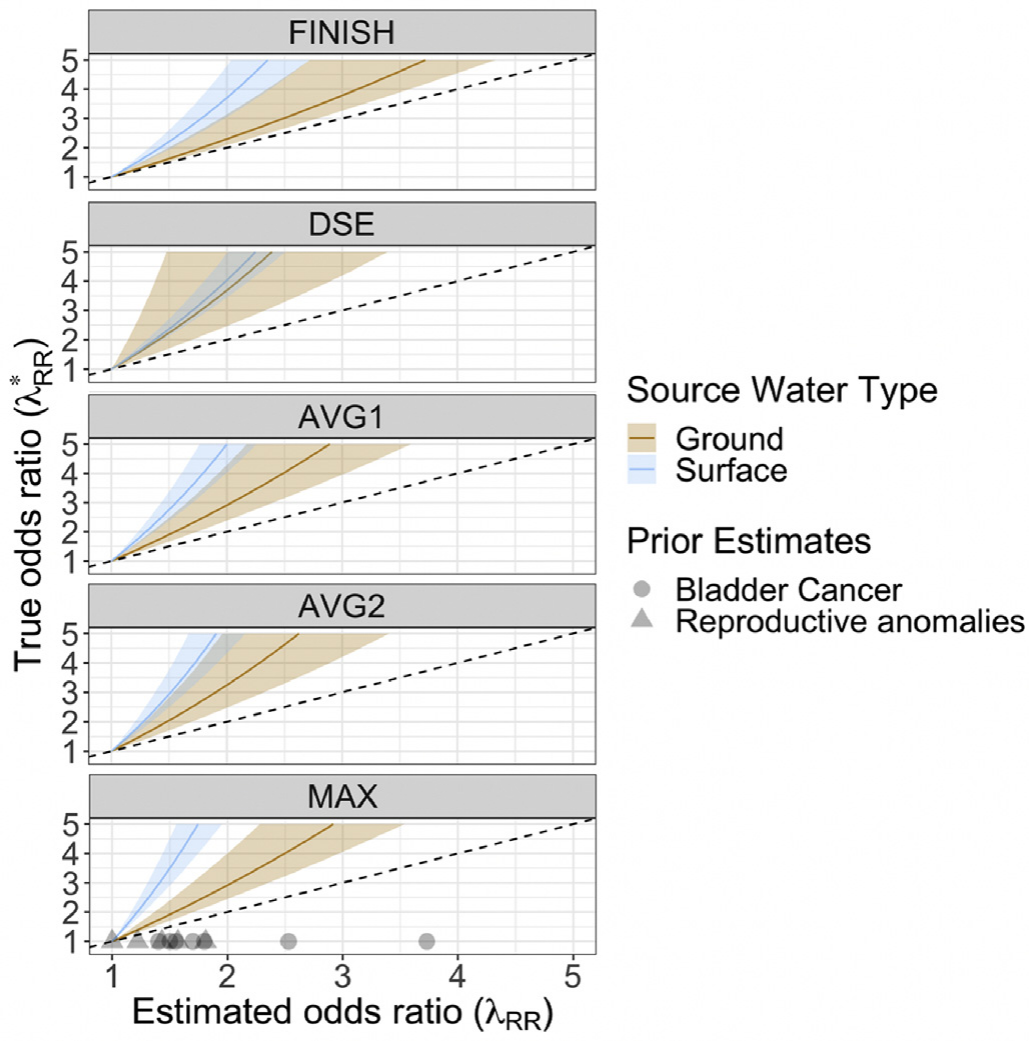
Risk ratio estimates resulting from true risk-ratios between 1 and 5 and estimated exposure misclassification, by source water type and sampling location. Note: dotted line plots y = x (i.e., no risk ratio estimation bias); sampling locations are ordered (top to bottom) by sample point distance from WTP; grey points on bottom panel give odds-ratio estimates from prior meta-analyses of DBP exposure and bladder cancer (Hrudey et al., 2015) and reproductive anomalies (Nieuwenhuijsen et al., 2009).

Odds ratio bias was estimated using the confusion matrix in Table 4 and Equation S(8). Fig. 4 provides the estimated mean odds ratios and 95% CI corresponding to “true” DBP-exposure odds ratios for each distribution system location for WTPs treating groundwater or surface water with chlorine during the summer. The results indicate that misclassification of HAN exposure from the use of THMs as a surrogate could lead to significant downward bias in odds ratio estimates for WTPs utilizing both surface water and groundwater, across each of the five sampling locations. The points in the bottom panel of Fig. 4 show selected odds-ratio estimates for DBP exposure’s effects on bladder cancer (Hrudey et al., 2015) and reproductive (i.e., congenital) anomalies (Nieuwenhuijsen et al., 2009). Prior odds ratio estimates are generally between 1 and 2, although values as high as 3.7 were found for certain subgroups at risk of bladder cancer (additional details provided in Text S3 and Table S9). Across the entire study sample, our estimates indicate that an estimated odds ratio of 2 may correspond to a true odds ratio of 4.6 and will be above 4.2 in 98% of cases.

The variation in odds ratio bias by source water type suggests that bias is often larger in surface water WTPs than groundwater WTPs. In finished effluent, an estimated odds ratio of 2 could correspond to a real odds ratio of 3.1–5.0 (95% CI) in surface water compared to 2.1–2.5 (95% CI) in groundwater. For surface water, bias also increases with distance from the water treatment plant. In the case of surface water sampled from the furthest (“MAX”) location category, a measured odds ratio of just 2 could correspond to a true odds ratio of 5.3–10.1.

## 4 Conclusions

Using THMs to measure DBP exposure implicitly assumes that THM concentrations are proportional to concentrations of more toxic DBPs in the mixture, and that this proportionality is robust to variables within water systems, such as distribution system residence time, and between water systems, such as differences in source waters. Using HANs as an example, this study employed statistical models to evaluate the assumption that THMs are proportional to more toxic DBP classes using a dataset of 9578 records from 412 large US water systems. Whether the data is pooled in a classic linear regression or hierarchically grouped by WTP in a multilevel regression, THMs only explain ∼30% of the variance in HANs and are poor predictors of HAN concentrations. The multilevel regression on the HAN:THM ratio demonstrated that THMs are not proportional to HANs within or between water systems, though much of the variance in the HAN:THM ratio (∼66%) was due to effects between water systems.

Four water system features (source water type, season, disinfectant sequence and distribution system location) accounted for 41% of the between-WTP variance in the HAN:THM ratio. Source water type had the largest magnitude effect, with groundwater exhibiting a 165% higher HAN:THM ratio than surface water under base-case conditions (chlorine-chlorine, finished effluent, summer). HAN:THM ratios were 38% higher during summer compared to winter in chlorinated surface water. Some disinfectant sequences were associated with a 20–30% change in the HAN:THM ratio relative to chlorine-chlorine disinfection. In chlorinated surface water during summer, HAN:THM ratios declined by 40% between finished effluents and maximum distribution system locations. In most cases, changes in THM concentrations drove differences in the HAN:THM ratio, while HANs were less affected by water system features.

A quantitative analysis of misclassification bias found that using high THM concentrations as an indicator for high HAN concentrations was associated with a 5.9% probability of false negatives and 5.9% probability of false positives. Across the study sample, an estimated odds ratio of 2 constructed based on THM concentrations may correspond to a “true” odds ratio of 4.6. The estimated odds ratio bias is greater in surface water than in groundwater, and increases with distance from the treatment plant. The results demonstrate that THMs are not a reliable surrogate for HANs, and the misclassification bias associated with the use of THMs to measure overall DBP exposure may significantly reduce the ability to discern associations between DBP exposure and adverse health outcomes.

As the >700 known DBPs account for less than half of the total organic halogen (TOX), identifying which DBPs drive adverse health effects is a significant challenge. Epidemiologic studies could aid this effort by targeting the analysis of more DBP classes, particularly high-toxicity classes such as HANs, through the use of existing datasets like the ICR and in new sampling campaigns. Measuring semi-volatile unregulated classes like HANs should not add excessive analytical burden to sampling efforts, as they can be extracted and analyzed using the same method as THMs. Furthermore, strategic data collection efforts are needed to identify whether HANs or other easily measured DBP classes are effective surrogates for exposure to a wide array of DBP classes. While previous survey efforts have focused on measuring unregulated DBPs in multiple water systems, this study demonstrates the need for better spatiotemporal resolution within water systems to evaluate the covariance of multiple classes with hydraulic residence time. Future DBP data collection efforts should be designed to achieve statistical significance with multilevel modeling techniques to accurately estimate variance between and within water systems for as many co-occurring DBP classes as possible.

## Funding

This work was supported by the 10.13039/100000001National Science Foundation Engineering Research Center for Re-Inventing the Nation’s Urban Water Infrastructure [grant number EEC-1028968]. The sponsor played no role in the study design, data collection and analysis, and decision to publish the manuscript. The findings, conclusions, and recommendations expressed in this manuscript are solely those of the authors.

The authors gratefully acknowledge Professors Meagan Mauter, Alexandria Boehm, and Richard Luthy of 10.13039/100005492Stanford University for providing valuable feedback that strengthened this manuscript.

## Declaration of competing interest

The authors declare that they have no known competing financial interests or personal relationships that could have appeared to influence the work reported in this paper.
