# Supplementary material for: Use of trihalomethanes as a surrogate for haloacetonitrile exposure introduces misclassification bias
Source: Water Res X. 2021 Jan 22;11:100089. doi: 10.1016/j.wroa.2021.100089 (PMC7851183; doi:10.1016/j.wroa.2021.100089)
Supplement: Multimedia component 1 [file mmc1.pdf]

Supplemental Information for:
Use of trihalomethanes as indicator of DBP exposure introduces potential for classification bias

Kirin E. Furst[1, 2 *], Jose Bolorinos[1], and William A. Mitch[1]

[1] Department of Civil and Environmental Engineering, Stanford University, 473 Via Ortega, Stanford, California 94305, United States

[2] Department of Civil, Environmental and Infrastructure Engineering, George Mason University, 4400 University Dr, Fairfax, VA 22030, United States

* Contact Information: Email: kfurst@gmu.edu, Phone: 703-993-3394

Table of Contents

Supplemental Text S1: Data processing 2

Supplemental Text S2: Sensitivity Analysis 4

Supplemental Text S3: Analysis of bias in risk ratio estimates 5

Additional References 7

Supplemental Figures 9

Supplemental Tables 11

Key to abbreviations in this document:

ICR: Information Collection Rule

WTP: Water treatment plant

PWS: Public water system

HANs: haloacetonitriles

THMs: trihalomethanes

Supplemental Text S1: Data processing

Data processing

The ICR AUX1 database was retrieved from the EPA data repository as a Microsoft Access file (.accdb) (US EPA, 2000) and extracted to Excel (.xlsx). Data was processed with Python v3.7 in the open-source scientific computing environment IPython v7.12 (Perez & Granger, 2007) using Pandas v1.0 (McKinney, 2012). Statistical analyses were conducted using Pandas and SciPy v1.18 (Virtanen et al., 2010). Figures were developed using Seaborn v0.10.1 (Waskom et al., 2018). Multilevel models were developed with Pymer4 v0.7.0, an open-source Python statistics library that interfaces with the lme4 package in the R statistical programming language (Jolly, 2018).

Screening

Source water

The ICR AUX 1 database contains a field for the source water type utilized by each WTP during the month of each sampling event. This field can take one of four categories: surface water (SW), groundwater (GW), mixed (MIX), and purchased/wholesale (PUR). MIX indicates multiple source water types with none exceeding 80% total flow. An additional field further specifies SW subcategories, of which one is groundwater under the influence of surface water (GI). For this study, GI was designated as a separate source water type, as examination of water quality parameters indicate it is distinct from other SW subcategories. A subset of ICR PWSs blended finished waters from multiple WTPs in the distribution system. These records were excluded, as it is impossible to ascertain which WTPs blended finished waters in what proportion. The source category field was empty (null) for 4% of records (n=384); all null entries were successfully recovered by examining other water resources information for that WTP during the month of sampling. Following screening, the records were 71% surface water (n=6772), 25% groundwater (n=2405), and 1–2% each of the remaining categories (Table S1A).

Season

ICR WTPs conducted sampling once per sample quarter over six quarters, defined in 3-month increments between July 2017 and December 2018. As one goal of the study was to evaluate the effects of season on the HAN:THM ratio, the month of sampling was used to re-sort records into quarters defined based on seasonal trends for North America, as indicated in Table S1A.

Distribution system residence time.

The distribution system sampling location was indicated by field EVNTTYPE, where the possible values are indicated in Table S1B along with the distribution of estimated RTs for each for each sampling location. The sampling location was used as a categorical proxy for distribution system residence time (RT) for two reasons: 1) The RT estimates were unreliable for many records, as indicated by a parameter rating the confidence level (high, medium, low) in the accuracy of the RT estimate, and whether blending occurred in the distribution system. Records with a high confidence rating were in the minority, and the reason for a low or medium rating (RT estimate, blending, or both) was not indicated. 2) The intermediate sampling locations (DSE, AVG1, AVG2) between finished effluent (FINISH) and the maximum RT (MAX) were not always sampled along the same distribution system branch; for example, AVG1 could be located between FINISH and MAX, while AVG2 is from a different branch.

Disinfectant sequence.

In the ICR AUX 1 database, for each WTP sampling period, the WTP_DIS field denotes the disinfectant(s) applied upstream of final residual application, and the DS_DIS field denotes the final disinfectant applied. Possible values for WTP_DIS are chlorine (CL2), chloramines (CLM), chlorine-chloramine (CL2-CLM), chlorine dioxide (CLX), ozone (O3), and null. Obolensky et al., (2007) replaced missing or erroneous WTP_DIS entries for 20% of records; unfortunately, details were not reported allowing us to emulate the

methods. For the present study, 19% (n=1,835) had null WTP_DIS entries. 14 null and 25 erroneous WTP_DIS entries were replaced (representing <1% of total records) following inspection of other WTP_DIS entries and process train information for that WTP. 1.2% of records (n=115) had null DS_DIS entries; all were replaced with CL2 or CLM following inspection of other DS_DIS entries and utility process information for that WTP, as well as relative concentrations of free and total chlorine residuals. WTP_DIS and DS_DIS entries were then concatenated for each record to create the disinfectant sequence. Two WTPs (20 records) had null WTP_DIS entries and CLM designated for DS_DIS; as mean THM and HAN concentrations were similar for between CLM and CLM-CLM, these records were reassigned as CLM-CLM for simplicity. The disinfectant sequences and number of records are provided in Table S1A.

Supplemental Text S2: Sensitivity Analysis

In many records, concentrations of one or more constituent species were below the method reporting limits (MRL) of 1.0 µg/L for each THM species and 0.5 µg/L for each HAN species. The fraction of left-censored records was 15% for HANs and 7.6% for THMs. For the results presented in the main text, left-censored measurements were replaced by half of the MRL for each DBP analyte (i.e., the "half-MRL" dataset). If the measurements for all 4 HANs in a sample were left-censored, the cumulative replacement value was 1.0 µg/L, and if all four THMs were censored, the cumulative replacement value was 2.0 µg/L.

The selection of replacement values for MRLs can affect the outcome of models using ICR data (Obolensky et al., 2005; Francis et al., 2009). The impact of the replacement value selection was tested on the multilevel models used in this study by comparing results when left-censored HAN and THM concentrations were replaced with the half-MRL values (e.g., the half-MRL dataset) versus the full-MRL values (e.g., the full-MRL dataset). The distributions of THM and HAN concentrations were shifted slightly higher in the full-MRL dataset. On a nM/nM basis, the median HAN:THM ratio was 0.16, compared to 0.14 for the half-MRL dataset, with a maximum of 1.72, compared to 2.2 for the half-MRL dataset; thus, the distribution of ratios was altered by the replacement of left-censored values.

The bivariate model with HANs regressed on THMs (Eq. 1) and the univariate model with HAN:THM as the outcome variable (Eq. 2) were fit to the half-MRL dataset and the full-MRL dataset, and to evaluate the change in estimated variances. For the bivariate model, the total variance was ~65% lower when fit to the full-MRL dataset compared to the half-MRL dataset (Table S2B). For the univariate model, the total variance was ~18% lower when fit to the full-MRL dataset (Table 4B) compared to the half-MRL dataset (Table 4A). Therefore, the bivariate model is more sensitive to left-censoring of concentrations, likely because the homogeneity of the 1,465 left-censored HAN measurements biases the bivariate model towards lower overall variance. In the univariate model with the HAN:THM ratio, left-censored HAN measurements are less influential because 57% (n=832) are divided by uncensored THM measurements. Only 6.6% of HAN:THM outcomes represent records where both HAN and THM measurements were censored, compared to 15% of HAN outcomes in the bivariate model.

The multilevel model with categorical predictors and interaction terms (Eq. 3) was rerun with the full-MRL dataset (Table S5C) for comparison with the half-MRL results reported in the main text (Table S5A, S5B). The systematic and residual variances were both almost 20% less than the estimates for the half-MRL dataset. However, the relative contributions of systematic and residual variance to the total variance remained consistent, with the systematic variance (0.233) higher than the residual variance (0.156). For the model fit to the full-MRL dataset, the conditional geometric mean HAN:THM ratio was 0.134, an 11.7% increase over the model run with the half-MRL dataset. The estimated effects of disinfectant sequence, season, and distribution system location were not substantially changed for surface water. However, some of the interactions of groundwater and groundwater under the influence with disinfectant sequence were smaller by up to 50%, reflecting the fact that groundwater-based source water types were more impacted by left-censoring. The results do not change the conclusions of the main text.

Supplemental Text S3: Analysis of bias in risk ratio estimates

As described in the main analysis, the health impacts of DBP-exposure are typically analyzed using logistic regression, which models the probability of a given health outcome Y as a function of a DBP exposure proxy (Z) and a set of other covariates W_j:

(S1) $$P(Y) = \frac{\exp(\alpha + \lambda Z + \sum_j \gamma_j W_j)}{1 + \exp(\alpha + \lambda Z + \sum_j \gamma_j W_j)}$$

Where $P(Y)$ is the probability of the adverse health outcome of interest and the coefficient λ measures the impact of proxied DBP exposure Z. In many studies, the exposure proxy Z is categorical (i.e., 1 if THM4 is within a given range and zero otherwise), such that the estimate λ is equivalent to the odds ratio for outcome Y for populations with observed exposure to DBPs relative to a base case – after controlling for the vector of W_j covariates:

(S2) $$\lambda = \frac{\frac{P(Y|Z,W)}{P(Y'|Z,W)}}{\frac{P(Y|Z',W)}{P(Y'|Z'W)}} = \frac{P(Y|Z,W)}{P(Y|Z',W)} \frac{P(Y'|Z',W)}{P(Y'|Z,W)}$$

In (S2) above, Y and Z denote the events where a person has disease outcome Y and observed exposure to DBPs, respectively (i.e. $Y = 1$ and $Z = 1$), and Y' and Z' are their complements (i.e. Y' and Z' denote events where $Y = 0$ and $Z = 0$), in which the disease outcome is negative and there is no observed exposure to DBPs; W is the vector of other covariates W_j.

Recall, that Z is based on high THM levels, and is only a proxy indicator of the true DBP exposure. A new binary variable is thus defined based on true DBP exposure X. This true exposure variable has complement X' denoting no actual DBP exposure (i.e. X = 0). The true odds ratio λ^* defining risk of DBP exposure is thus:

(S3) $$\lambda^* = \frac{P(Y|X,W)}{P(Y|X',W)} \frac{P(Y'|X',W)}{P(Y'|X,W)}$$

Several assumptions are made to estimate bias in the estimate λ of the true odds ratio λ^*. First, health condition Y is assumed to have a low prevalence rate, such that $P(Y'|X',W)$ and $P(Y'|X,W)$ are both very close to 1 and the odds ratios given in (S2) and (S3) are very similar to risk ratios (i.e. $\frac{P(Y|Z,W)}{P(Y|Z',W)}$ and $\frac{P(Y|X,W)}{P(Y|X',W)}$):

(S4) $$\lambda^* = \frac{P(Y|X,W)}{P(Y|X',W)} \frac{P(Y'|X',W)}{P(Y'|X,W)} \approx \frac{P(Y|X,W)}{P(Y|X',W)} = \lambda^*_{RR}$$

The assumption of low prevalence is justifiable for both bladder cancer and congenital anomalies. One review of bladder cancer found a high-end prevalence rate of 185 per 100,000 males in Italy (Crocetti et al., 2013), corresponding to the probability of an Italian male not getting bladder cancer, $P(Y')$ of 99.8%. The prevalence of any congenital anomaly in Europe was found to be 2.4%, but individual anomaly classes are rarer (the most common anomaly is 0.65%, corresponding to a $P(Y')$ of 99.35%.) (Dolk et al., 2010).

Misclassification of true exposure X is also assumed to be determined by water quality parameters that are independent of covariates W or health outcome Y (i.e. misclassification is non-differential). Estimation of odds ratio bias for differential exposure misclassification with respect to Y or W is left as a topic for future study. Assuming nondifferential misclassification of X, $P(Y|Z,W)$ and $P(Y|Z',W)$ in (5) above to be factored as follows:

(S5) $$P(Y|Z,W) = \frac{P(Y|X,W)P(X,Z) + P(Y|X',W)P(X',Z)}{P(Z)}$$

(S6) $$P(Y|Z',W) = \frac{P(Y|X,W)P(X,Z')+P(Y|X',W)P(X',Z')}{P(Z')}$$

Note that (S5) and (S6) also assume that Z (THM indicator) affects Y (disease outcome) only through its association with true exposure X (HAN indicator). Substituting the expressions for $P(Y|Z,W)$ and $P(Y|Z',W)$ in (S5) and (S6) above into an estimated risk ratio from observed DBP exposure (Z), we get the following:

(S7) $$\lambda_{RR} = \frac{P(Z')}{P(Z)}\frac{P(Y|X,W)P(X,Z)+P(Y|X',W)P(X',Z)}{P(Y|X,W)P(X,Z')+P(Y|X',W)P(X',Z')}$$

Where $P(X,Z), P(X',Z), P(X,Z'), P(X',Z')$ are probabilities of true positive, false positive, false negative, and true negative DBP exposure classification, respectively, from using THMs as an indicator variable. (S7) can then be expressed in terms of the true risk-ratio λ_{RR}^* and these probabilities, which can be computed empirically from ICR data:

(S8) $$\lambda_{RR} = \frac{P(Z')}{P(Z)}\left(\frac{\lambda_{RR}^* P(X,Z)}{\lambda_{RR}^* P(X,Z')+P(X',Z')} + \frac{\lambda_{RR}^{*-1} P(X',Z)}{\lambda_{RR}^{*-1} P(X',Z')+P(X,Z')}\right)$$

Table S9 shows odds-ratio ratio estimates from meta-analyses of the risks of bladder cancer (Hrudey et al., 2015) and reproductive (congenital) anomalies (Nieuwenhuijsen et al., 2009) from DBP-exposure, which were used as a reference point for gauging the magnitude of odds-ratio bias likely to result from DBP exposure misclassification. The THM concentration threshold used as an indicator in this study is the 90th percentile concentration in the ICR dataset (74.3 μg/L). In the cited studies, the range of THM thresholds varies widely (20-130 μg/L), as does the range of baseline THM concentrations (1-60 μg/L). Given this, it is important to note that these estimates are included in our analysis purely for contextual purposes and are not intended to be used to infer actual odds-ratio bias.

Additional References

Bove F., Shim Y., Zeitz P., 2002. Drinking water contaminants and adverse pregnancy outcomes: a review. Environ Health Perspect. 110 Suppl 1(Suppl 1):61-74. https://doi.org/10.1289/ehp.02110s161

Hwang B. F., Jaakkola J. J., 2003. Water chlorination and birth defects: a systematic review and meta-analysis. Arch Environ Health. 58(2):83-91. https://doi.org/10.3200/AEOH.58.2.83-91.

Cantor, K. P., Villanueva, C. M., Silverman, D. T., Figueroa, J. D., Real, F. X., Garcia-Closas, M., Malats, N., Chanock, S., Yeager, M., Tardon, A., Garcia-Closas, R., Serra, C., Carrato, A., Castaño-Vinyals, G., Samanic, C., Rothman, N., & Kogevinas, M., 2010. Polymorphisms in GSTT1, GSTZ1, and CYP2E1, disinfection by-products, and risk of bladder cancer in Spain. Environmental health perspectives. 118(11), 1545–1550. https://doi.org/10.1289/ehp.1002206

Chevrier, C., Junod, B., & Cordier, S. (2004). Does Ozonation of Drinking Water Reduce the Risk of Bladder Cancer Epidemiology. 15(5), 605-614. Retrieved October 12, 2020, from http://www.jstor.org/stable/20485960

Chisholm K., Cook A., Bower C., Weinstein P., 2008. Risk of birth defects in Australian communities with high brominated disinfection by-product levels. Environ Health Perspect. 116:1267–1273. https://doi.org/10.1289/ehp.10980

Crocetti, E., De Angelis, R., Buzzoni, C. *et al.,* 2013. Cancer prevalence in United States, Nordic Countries, Italy, Australia, and France: an analysis of geographic variability. *Br J Cancer* **109,** 219–228. https://doi.org/10.1038/bjc.2013.311

Dolk H., Loane M., Garne E., 2010. The Prevalence of Congenital Anomalies in Europe. In: Posada de la Paz M., Groft S. (eds) Rare Diseases Epidemiology. Advances in Experimental Medicine and Biology, vol 686. Springer, Dordrecht. https://doi.org/10.1007/978-90-481-9485-8_20

Francis, R.A., Small, M.J., VanBriesen, J.M., 2009. Multivariate distributions of disinfection by-products in chlorinated drinking water. Water Res. 43, 3453–3468. https://doi.org/10.1016/j.watres.2009.05.008

Hrudey, S.E., Backer, L.C., Humpage, A.R., Krasner, S.W., Michaud, D.S., Moore, L.E., Singer, P.C., Stanford, B.D., 2015. Evaluating Evidence for Association of Human Bladder Cancer with Drinking-Water Chlorination Disinfection By-Products. J. Toxicol. Environ. Heal. - Part B Crit. Rev. 18, 213–241. https://doi.org/10.1080/10937404.2015.1067661

Jolly, E., 2018. Pymer4: Connecting R and Python for Linear Mixed Modeling. J. Open Source Softw. 3, 862. https://doi.org/10.21105/joss.00862

King W. D., Marrett L. D., 1996. Case-control study of bladder cancer and chlorination by-products in treated water (Ontario, Canada). Cancer Causes Control. 7(6):596-604. https://doi.org/10.1007/BF00051702.

McKinney, W., 2012. Python for data analysis: Data wrangling with Pandas, NumPy, and IPython. O'Reilly Media, Inc.

Nieuwenhuijsen MJ, Toledano MB, Bennett J, Best N, Hambly P, de Hoogh K, et al. 2008. Chlorination disinfection by-products and risk of congenital anomalies in England and Wales. Environ Health Perspect. 116:216–222. https://doi.org/10.1289/ehp.10636

Nieuwenhuijsen, M.J., Martinez, D., Grellier, J., Bennett, J., Best, N., Iszatt, N., Vrijheid, M., Toledano, M.B., 2009. Chlorination Disinfection By-Products in Drinking Water and Congenital Anomalies: Review and Meta-Analyses. Environ. Health Perspect. 117, 1486–1493. https://doi.org/10.1289/ehp

Obolensky, A., Singer, P.C., 2005. Halogen substitution patterns among disinfection byproducts in the information collection rule database. Environ. Sci. Technol. 39, 2719–2730. https://doi.org/10.1021/es0489339

Pérez, F., Granger, B.E., 2007. IPython: A system for interactive scientific computing. Comput. Sci. Eng. 9, 21–29. https://doi.org/10.1109/MCSE.2007.53

Villanueva C. M., Cantor K. P., Grimalt J.O., Malats N., Silverman D., Tardon A., Garcia-Closas R., Serra C., Carrato A., Castaño-Vinyals G., Marcos R., Rothman N., Real F.X., Dosemeci M., Kogevinas M., 2007. Bladder cancer and exposure to water disinfection by-products through ingestion, bathing, showering, and swimming in pools. Am J Epidemiol. 165(2):148-56. https://doi.org/10.1093/aje/kwj364.

Virtanen, P., Gommers, R., Oliphant, T.E., Haberland, M., Reddy, T. et al., 2020. SciPy 1.0: fundamental algorithms for scientific computing in Python. Nat. Methods 17, 261–272. https://doi.org/10.1038/s41592-019-0686-2

Waskom, M., Botvinnik, O., Hobson, P., Warmenhoven, J., Cole, J.B., Halchenko, Y., Vanderplas, J., Hoyer, S., Villalba, S., Quintero, E., others, 2014. Seaborn: statistical data visualization. URL https//seaborn. pydata. org/(visited 2017-05-15). https://doi.org/https://doi.org/10.5281/zenodo.1313201

Supplemental Figures

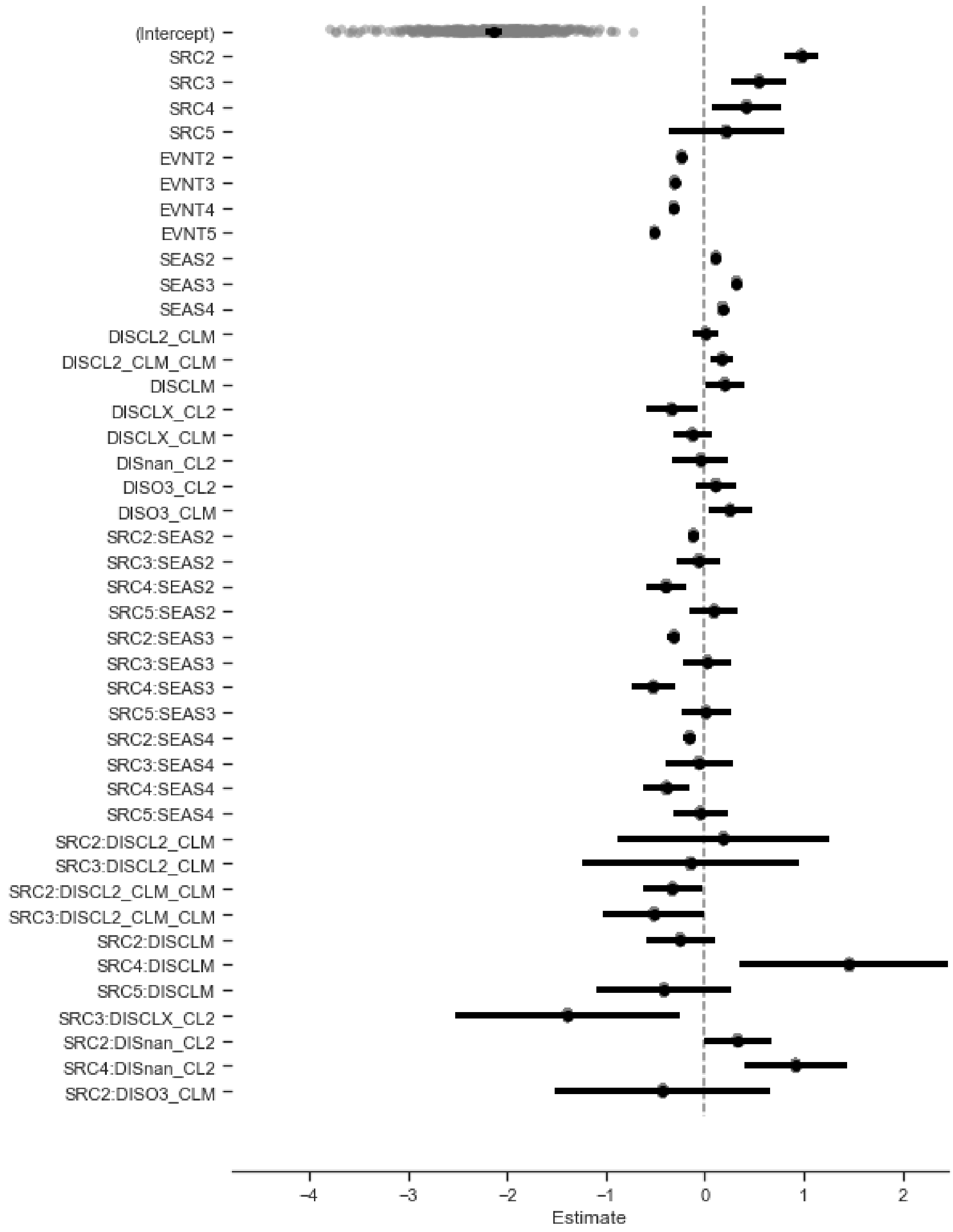

Figure S1: Plot showing the fixed and random effects on the conditional expected mean (intercept), and fixed effects of all predictor variables on the expected mean (with 95% confidence intervals) on a logarithmic scale.

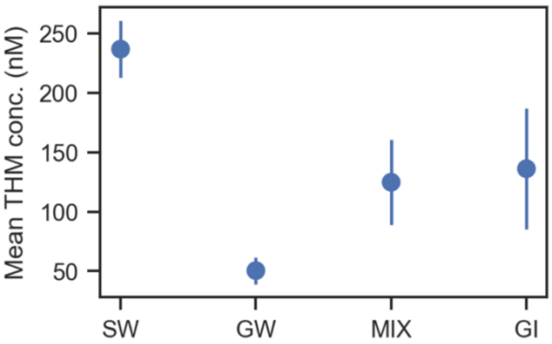

A)

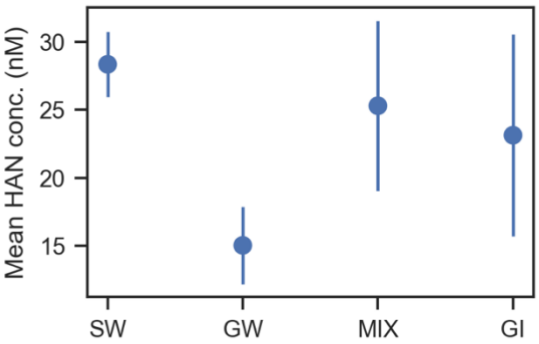

B)

Figure S2: Estimated geometric mean of A) THM and B) HAN concentrations (nM) by source water, in finished effluent disinfected with CL2-CL2 in the summer (i.e., the base-case conditions), estimated with random intercept multilevel models. For untransformed coefficient estimates and statistical significance, refer to Tables D.8 and D.10 respectively. (Note: effect of MIX and GI on HAN concentrations not statistically significant relative to GW.)

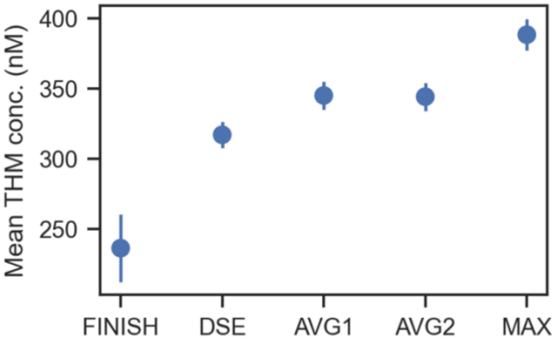

A)

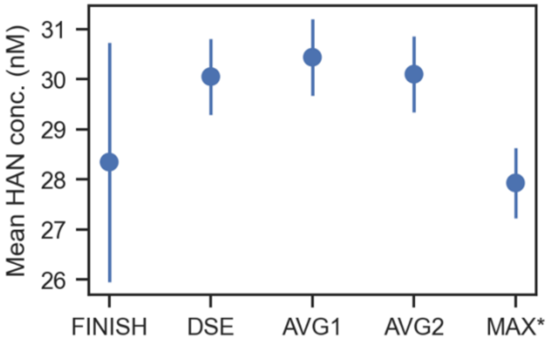

B)

Figure S3: Estimated geometric mean of A) THM and B) HAN concentrations (nM) by sample location, in surface water disinfected with CL2-CL2 in the summer (i.e., the base-case conditions), estimated with random intercept multilevel models. For untransformed coefficient estimates and statistical significance, refer to Tables D.8 and D.10 respectively. (Note: effect of MAX on HAN concentrations not statistically significant relative to FINISH.)

Supplemental Tables

Key for Supplemental Tables:

Significance is determined based on a 95% confidence level (p-value<0.05) unless indicated otherwise:

.	On the threshold of significance to a 95% confidence level
*	Significant to a 95% confidence level
**	Significant to a 99% confidence level
***	Significant to a 99.9% confidence level

Random effects table headings:

Var	Variance
Std	Standard deviation

Fixed effects table headings:

ci	Confidence interval
SE	Standard error
DF	Degrees of freedom
T-stat	T-statistic
P-val	p-value (used to determine significance level)
Sig	Significance level

Table S1A – Categorical predictor variables, with coding used in the model and number of records per category.

Predictor	Categories	Modeling code	# records
Source water type (SRC)	Surface water (SW)	SRC1	6772
	Groundwater (GW)	SRC2	2405
	Mixed (MIX)	SRC3	119
	GW under the influence of SW (GI)	SRC4	166
	Purchased/wholesale (PUR)	SRC5	125
DS sampling location (EVNT)	FINISH	EVNT1	1962
	DSE	EVNT2	1922
	AVG1	EVNT3	1972
	AVG2	EVNT4	1948
	MAX	EVNT5	1967
Season (SEAS)	Summer (Jun-Aug	SEAS1	2652
	Autumn (Sept-Nov)	SEAS2	3249
	Winter (Dec-Feb)	SEAS3	2009
	Spring (Mar-May)	SEAS4	1677
Disinfectant sequence (DIS)	Cl2-CL2	DISCL2_CL2	4486
	CL2	DISnan_CL2	1628
	Cl2-CLM-CLM	DISCL2_CLM_CLM	1791
	CLM*	DISCLM_CLM	478
	CL2-CLM	DISCL2_CLM	423
	CLX-CL2	DISCLX_CL2	213
	CLX-CLM	DISCLX_CLM	196
	O3-CL2	DISO3_CL2	121
	O3-CLM	DISO3_CLM	251

Table S1B – Statistics of distribution system measurements by sampling location.

DS Sampling location (EVNT)	Modeling code	mean	std	min	25%	50%	75%	max
Finished effluent	FINISH	NA	NA	NA	NA	NA	NA	NA
Distribution system equivalent*	DSE	16.5	18.2	1.0	3.0	10.0	24.0	120.0
Average RT	AVG1	27.2	34.1	1.0	6.0	17.0	35.0	240.0
Average RT	AVG2	31.4	48.8	1.0	6.0	20.0	36.0	960.0
Maximum RT	MAX	68.7	88.0	1.0	18.0	48.0	84.0	999.0

Table notes: Distribution system equivalent (DSE) is a real distribution system sample collected from an intermediate location, which was typically closer to the entrance to the distribution system compared to AVG1 and AVG2.

Table S2A – Variances (random effects) and coefficient estimates (fixed effects) for the bivariate multilevel model with HANs (log–nM) as the outcome variable and THMs (log-nM) as a predictor variable.

Random effect	Var	Std						
WTP intercepts	0.087	0.295						
WTP slopes	0.008	0.088						
Residual	0.128	0.357						
**Coefficient**	**Estimate**	**2.5_ci**	**97.5_ci**	**SE**	**DF**	**T-stat**	**P-val**	**Sig**
Intercept	1.334	1.257	1.411	0.039	574.803	34.078	4.38E-140	***
Slope	0.349	0.333	0.365	0.008	1148.152	41.509	9.29E-231	***

Table notes: Confidence intervals, standard error, and significance are all on the estimate of the regression coefficients, i.e., the population means, and not the dispersal of the estimated coefficients for the 412 individual linear regressions.

Table S2B – Sensitivity analysis: Variances (random effects) and coefficient estimates (fixed effects) for the bivariate multilevel model with HANs (log–nM) as the outcome variable and THMs (log-nM) as a predictor variable, fit to the full-MRL dataset.

Random effect	Var	Std						
WTP intercepts	0.030	0.173						
WTP slopes	0.004	0.067						
Residual	0.076	0.276						
**Coefficient**	**Estimate**	**2.5_ci**	**97.5_ci**	**SE**	**DF**	**T-stat**	**P-val**	**Sig**
Intercept	1.820	1.757	1.883	0.032	850.972	56.329	0.0	***
THM4_log	0.294	0.280	0.308	0.007	1803.220	42.327	0.0	***

Table notes: Confidence intervals, standard error, and significance are all on the estimate of the regression coefficients, i.e., the population means, and not the dispersal of the estimated coefficients for the 412 individual linear regressions.

Table S3 – Variances (random effects) and coefficient estimates (fixed effects) for the univariate multilevel model with HANs (log–nM) as the outcome variable.

Random effect	Var	Std						
WTP intercepts	0.476	0.690						
Residual	0.161	0.402						
**Coefficient**	**Estimate**	**2.5_ci**	**97.5_ci**	**SE**	**DF**	**T-stat**	**P-val**	**Sig**
Intercept	3.147	3.080	3.215	0.034	410.324	91.819	0.0	***

Table notes: Confidence intervals, standard error, and significance are all on the estimate of the regression coefficients, i.e., the population means, and not the dispersal of the estimated coefficients for the 412 individual linear regressions.

Table S4A – Variances (random effects) and coefficient estimates (fixed effects) for the univariate multilevel model with HAN:THM ratio as the outcome variable (log–nM/nM).

Random effect	Var	Std						
WTP intercepts	0.480	0.693						
Residual	0.249	0.499						
**Coefficient**	**Estimate**	**2.5_ci**	**97.5_ci**	**SE**	**DF**	**T-stat**	**P-val**	**Sig**
Intercept	-1.9819	-2.0498	-1.9141	0.0346	411.7739	-57.2711	0.0	***

Table notes: Confidence intervals, standard error, and significance are all on the estimate of the regression coefficients, i.e., the population means, and not the dispersal of the estimated coefficients for the 412 individual linear regressions.

Table S4B – Sensitivity analysis: Variances (random effects) and coefficient estimates (fixed effects) for the univariate multilevel model with HAN:THM ratio as the outcome variable (log–nM/nM), fit to the full-MRL dataset.

Random effect	Var	Std						
WTP	0.425	0.652						
Residual	0.187	0.432						
**Coefficient**	**Estimate**	**2.5_ci**	**97.5_ci**	**SE**	**DF**	**T-stat**	**P-val**	**Sig**
Intercept	-1.843	-1.906	-1.779	0.032	411.264	-56.737	0.0	***

Table notes: Confidence intervals, standard error, and significance are all on the estimate of the regression coefficients, i.e., the population means, and not the dispersal of the estimated coefficients for the 412 individual linear regressions.

Table S5A – Variances (random effects) and coefficient estimates (fixed effects) for the multilevel random intercept model with HAN:THM ratio as the outcome variable (log–nM/nM) and categorical predictor variables.

Random effects	Var	Std						
WTP intercepts	0.283	0.532						
Residual	0.212	0.460						
**Fixed effects**	**Estimate**	**2.5_ci**	**97.5_ci**	**SE**	**DF**	**T-stat**	**P-val**	**Sig**
(Intercept)	-2.133	-2.210	-2.055	0.039	686.164	-54.167	0.000	***
SRC2	0.976	0.798	1.154	0.091	781.053	10.767	0.000	***
SRC3	0.548	0.273	0.822	0.140	8418.078	3.912	0.000	***
SRC4	0.423	0.072	0.774	0.179	6100.803	2.364	0.018	*
SRC5	0.216	-0.366	0.798	0.297	577.937	0.728	0.467	
EVNT2	-0.235	-0.264	-0.205	0.015	9134.785	-15.683	0.000	***
EVNT3	-0.307	-0.336	-0.278	0.015	9135.273	-20.646	0.000	***
EVNT4	-0.315	-0.344	-0.286	0.015	9135.107	-21.142	0.000	***
EVNT5	-0.511	-0.540	-0.481	0.015	9135.387	-34.329	0.000	***
SEAS2	0.112	0.083	0.142	0.015	9208.818	7.561	0.000	***
SEAS3	0.323	0.290	0.356	0.017	9230.221	18.993	0.000	***
SEAS4	0.180	0.146	0.215	0.018	9243.595	10.188	0.000	***
DISCL2_CLM	0.010	-0.126	0.146	0.069	5107.905	0.145	0.885	
DISCL2_CLM_CLM	0.176	0.059	0.293	0.060	1055.646	2.949	0.003	**
DISCLM	0.202	0.011	0.392	0.097	2162.100	2.075	0.038	*
DISCLX_CL2	-0.336	-0.598	-0.075	0.133	1454.906	-2.519	0.012	*
DISCLX_CLM	-0.126	-0.318	0.067	0.098	2947.433	-1.281	0.200	
DISnan_CL2	-0.041	-0.326	0.244	0.145	1047.154	-0.280	0.780	
DISO3_CL2	0.112	-0.090	0.315	0.103	5432.113	1.086	0.277	
DISO3_CLM	0.256	0.036	0.476	0.112	2521.935	2.282	0.023	*

Table key: ci: 95% confidence interval; SE: standard error; DF: degrees freedom; Sig, significance, with * indicating 95% confidence level, ** indicating 99% confidence level, and *** indicating above the 99.9% confidence level.

Table S5B – Coefficient estimates for interaction terms in the multilevel random intercept model with HAN:THM ratio as the outcome variable (log–nM/nM) and categorical predictor variables.

Fixed effects	Estimate	2.5_ci	97.5_ci	SE	T-stat	P-val	Sig
SRC2:DISCL2_CLM	0.191	-0.886	1.267	0.549	0.347	0.729	
SRC3:DISCL2_CLM	-0.143	-1.239	0.953	0.559	-0.256	0.798	
SRC2:DISCL2_CLM_CLM	-0.326	-0.622	-0.031	0.151	-2.164	0.031	*
SRC3:DISCL2_CLM_CLM	-0.514	-1.026	-0.003	0.261	-1.971	0.049	*
SRC2:DISCLM	-0.249	-0.599	0.100	0.178	-1.397	0.163	
SRC4:DISCLM	1.461	0.346	2.577	0.569	2.567	0.011	*
SRC5:DISCLM	-0.413	-1.089	0.263	0.345	-1.196	0.232	
SRC3:DISCLX_CL2	-1.389	-2.520	-0.258	0.577	-2.408	0.016	*
SRC2:DISnan_CL2	0.333	-0.014	0.680	0.177	1.880	0.060	.
SRC4:DISnan_CL2	0.918	0.405	1.431	0.262	3.505	0.000	***
SRC2:DISO3_CLM	-0.424	-1.514	0.667	0.556	-0.762	0.447	
SRC2:SEAS2	-0.116	-0.172	-0.060	0.029	-4.051	0.000	***
SRC3:SEAS2	-0.061	-0.279	0.157	0.111	-0.548	0.584	
SRC4:SEAS2	-0.391	-0.588	-0.193	0.101	-3.875	0.000	***
SRC5:SEAS2	0.096	-0.148	0.340	0.124	0.772	0.440	
SRC2:SEAS3	-0.310	-0.375	-0.244	0.033	-9.313	0.000	***
SRC3:SEAS3	0.026	-0.211	0.263	0.121	0.218	0.827	
SRC4:SEAS3	-0.521	-0.734	-0.307	0.109	-4.772	0.000	***
SRC5:SEAS3	0.012	-0.239	0.263	0.128	0.091	0.927	
SRC2:SEAS4	-0.155	-0.222	-0.087	0.034	-4.498	0.000	***
SRC3:SEAS4	-0.058	-0.399	0.283	0.174	-0.334	0.738	
SRC4:SEAS4	-0.388	-0.616	-0.160	0.116	-3.333	0.001	***
SRC5:SEAS4	-0.045	-0.319	0.229	0.140	-0.324	0.746	

Table key: ci: 95% confidence interval; SE: standard error; DF: degrees freedom; Sig, significance, with * indicating 95% confidence level, ** indicating 99% confidence level, and *** indicating above the 99.9% confidence level.

Table S5C – Sensitivity analysis of the impact of left-censoring on the multilevel model with with HAN:THM ratio as the outcome variable (log–nM/nM) and categorical predictor variables, fit to the full-MRL dataset.

Random effects	Var	Std						
WTP intercepts	0.233	0.483						
Residual	0.156	0.395						
**Fixed effects**	**Estimate**	**2.5_ci**	**97.5_ci**	**SE**	**DF**	**T-stat**	**P-val**	**Sig**
(Intercept)	-2.013	-2.082	-1.944	0.035	674.823	-57.241	0.000	***
SRC2	1.016	0.858	1.175	0.081	807.258	12.592	0.000	***
SRC3	0.555	0.318	0.791	0.121	8425.924	4.594	0.000	***
SRC4	0.488	0.184	0.792	0.155	6577.808	3.148	0.002	**
SRC5	0.129	-0.393	0.650	0.266	568.689	0.483	0.629	
EVNT2	-0.216	-0.242	-0.191	0.013	9132.747	-16.871	0.000	***
EVNT3	-0.289	-0.314	-0.264	0.013	9133.164	-22.692	0.000	***
EVNT4	-0.290	-0.315	-0.264	0.013	9133.022	-22.658	0.000	***
EVNT5	-0.451	-0.476	-0.426	0.013	9133.276	-35.406	0.000	***
SEAS2	0.116	0.091	0.141	0.013	9201.304	9.112	0.000	***
SEAS3	0.316	0.287	0.344	0.015	9221.349	21.655	0.000	***
SEAS4	0.174	0.144	0.203	0.015	9234.130	11.454	0.000	***
DISCL2_CLM	0.036	-0.082	0.154	0.060	5582.129	0.602	0.547	
DISCL2_CLM_CLM	0.154	0.050	0.258	0.053	1136.309	2.906	0.004	**
DISCLM	0.243	0.076	0.409	0.085	2333.246	2.850	0.004	**
DISCLX_CL2	-0.262	-0.492	-0.032	0.117	1607.515	-2.229	0.026	*
DISCLX_CLM	-0.086	-0.254	0.083	0.086	3178.794	-0.998	0.318	
DISnan_CL2	0.010	-0.242	0.263	0.129	1117.413	0.080	0.936	
DISO3_CL2	0.125	-0.051	0.301	0.090	5843.457	1.392	0.164	
DISO3_CLM	0.287	0.095	0.480	0.098	2819.979	2.927	0.003	**
SRC2:SEAS2	-0.105	-0.153	-0.057	0.025	9207.954	-4.267	0.000	***
SRC3:SEAS2	-0.049	-0.236	0.138	0.095	9146.863	-0.512	0.609	
SRC4:SEAS2	-0.310	-0.479	-0.140	0.086	9285.800	-3.585	0.000	***
SRC5:SEAS2	0.060	-0.149	0.269	0.107	9169.218	0.567	0.571	
SRC2:SEAS3	-0.297	-0.353	-0.242	0.029	9225.907	-10.437	0.000	***
SRC3:SEAS3	0.034	-0.169	0.238	0.104	9268.478	0.332	0.740	
SRC4:SEAS3	-0.383	-0.566	-0.200	0.094	9181.961	-4.095	0.000	***
SRC5:SEAS3	-0.032	-0.247	0.183	0.110	9190.980	-0.291	0.771	
SRC2:SEAS4	-0.141	-0.198	-0.083	0.030	9228.665	-4.763	0.000	***
SRC3:SEAS4	-0.052	-0.344	0.241	0.149	9142.360	-0.346	0.729	

SRC4:SEAS4	-0.401	-0.597	-0.206	0.100	9173.455	-4.020	0.000	***
SRC5:SEAS4	-0.053	-0.287	0.182	0.120	9194.335	-0.440	0.660	
SRC2:DISCL2_CLM	0.078	-0.897	1.053	0.497	389.188	0.156	0.876	
SRC3:DISCL2_CLM	-0.275	-1.265	0.715	0.505	421.488	-0.545	0.586	
SRC2:DISCL2_CLM_CLM	-0.431	-0.691	-0.171	0.133	1729.220	-3.251	0.001	**
SRC3:DISCL2_CLM_CLM	-0.520	-0.962	-0.078	0.225	7511.768	-2.307	0.021	*
SRC2:DISCLM	-0.296	-0.603	0.010	0.156	2291.996	-1.897	0.058	.
SRC4:DISCLM	1.134	0.127	2.141	0.514	436.547	2.207	0.028	*
SRC5:DISCLM	-0.294	-0.895	0.307	0.307	869.042	-0.960	0.337	
SRC3:DISCLX_CL2	-1.377	-2.397	-0.357	0.520	452.910	-2.646	0.008	**
SRC2:DISnan_CL2	0.182	-0.126	0.490	0.157	1008.832	1.160	0.246	
SRC4:DISnan_CL2	0.583	0.138	1.028	0.227	5430.951	2.565	0.010	*
SRC2:DISO3_CLM	-0.566	-1.553	0.420	0.503	399.929	-1.125	0.261	

Table key: ci: 95% confidence interval; SE: standard error; DF: degrees freedom; Sig, significance, with * indicating 95% confidence level, ** indicating 99% confidence level, and *** indicating above the 99.9% confidence level.

Table S6 – ANOVA results for the multilevel random intercept model with HAN:THM ratio as the outcome variable (log–nM/nM) and categorical predictor variables.

Predictor	SS	MS	NumDF	DenomDF	F-stat	P-value	Sig
SRC	7.592	1.898	4	544.672	8.940	5.352e-07	***
EVNT	259.543	64.885	4	9134.476	305.629	3.882e-247	***
SEAS	4.192	1.397	3	9222.056	6.582	1.934-04	***
DIS	5.676	0.709	8	737.100	3.341	9.080e-04	***
SRC:DIS	7.492	0.681	11	716.825	3.208	2.814e-04	***
SRC:SEAS	23.453	1.954	12	9219.638	9.206	6.331e-18	***

Table key: SS, sum of squares; MS, sum of squares divided by the degrees of freedom; DF, degrees of freedom; Sig, significance, with * indicating 95% confidence level, ** indicating 99% confidence level, and *** indicating above the 99.9% confidence level.

Table S7 – Variances (random effects) and estimated coefficients (fixed effects) for the multilevel model with HAN concentration as the outcome variable (log–nM).

Random effects	Var	Std						
WTP intercepts	0.398	0.631						
Residual	0.157	0.396						
**Fixed effects**	**Estimate**	**2.5_ci**	**97.5_ci**	**SE**	**DF**	**T-stat**	**P-val**	**Sig**
(Intercept)	3.345	3.260	3.429	0.043	624.856	77.830	0.000	***
SRC2	-0.635	-0.822	-0.447	0.096	961.754	-6.617	0.000	***
SRC3	-0.114	-0.358	0.131	0.125	8351.934	-0.912	0.362	
SRC4	-0.203	-0.519	0.113	0.161	8424.328	-1.261	0.207	
SRC5	0.243	-0.405	0.892	0.331	530.295	0.735	0.463	
EVNT2	0.058	0.033	0.084	0.013	9122.407	4.540	0.000	***
EVNT3	0.071	0.046	0.096	0.013	9122.570	5.576	0.000	***
EVNT4	0.060	0.035	0.085	0.013	9122.508	4.683	0.000	***
EVNT5	-0.015	-0.040	0.010	0.013	9122.670	-1.156	0.248	
SEAS2	-0.029	-0.054	-0.004	0.013	9170.034	-2.240	0.025	*
SEAS3	-0.105	-0.134	-0.076	0.015	9184.250	-7.179	0.000	***
SEAS4	-0.043	-0.073	-0.013	0.015	9193.795	-2.815	0.005	**
DISCL2_CLM	-0.209	-0.333	-0.085	0.063	7669.740	-3.307	0.001	***
DISCL2_CLM_CLM	0.042	-0.077	0.161	0.061	1691.950	0.691	0.490	
DISCLM	-0.395	-0.578	-0.212	0.093	3371.409	-4.236	0.000	***
DISCLX_CL2	-0.270	-0.527	-0.013	0.131	2661.482	-2.059	0.040	*
DISCLX_CLM	-0.498	-0.680	-0.317	0.093	4488.945	-5.381	0.000	***
DISnan_CL2	-0.007	-0.297	0.283	0.148	1603.468	-0.048	0.962	
DISO3_CL2	-0.292	-0.476	-0.108	0.094	7681.518	-3.108	0.002	**
DISO3_CLM	-0.375	-0.583	-0.167	0.106	4663.158	-3.534	0.000	***

Table key: ci: 95% confidence interval; SE: standard error; DF: degrees freedom; Sig, significance, with * indicating 95% confidence level, ** indicating 99% confidence level, and *** indicating above the 99.9% confidence level. *Table notes:* Estimates for interaction coefficients not included for brevity.

Table S8 – Variances (random effects) and estimated coefficients (fixed effects) for the multilevel model with THM concentration as the outcome variable (log–nM).

Random effects	Var	Std						
WTP intercepts	0.591	0.769						
Residual	0.206	0.454						
**Fixed effects**	**Estimate**	**2.5_ci**	**97.5_ci**	**SE**	**DF**	**T-stat**	**P-val**	**Sig**
(Intercept)	5.466	5.365	5.567	0.052	626.119	105.886	0.000	***
SRC2	-1.546	-1.769	-1.322	0.114	1025.219	-13.550	0.000	***
SRC3	-0.640	-0.923	-0.358	0.144	8340.499	-4.444	0.000	***
SRC4	-0.553	-0.918	-0.188	0.186	8736.768	-2.971	0.003	**
SRC5	0.093	-0.690	0.875	0.399	532.755	0.232	0.817	
EVNT2	0.293	0.264	0.322	0.015	9128.667	19.869	0.000	***
EVNT3	0.378	0.349	0.407	0.015	9128.785	25.794	0.000	***
EVNT4	0.375	0.346	0.404	0.015	9128.737	25.528	0.000	***
EVNT5	0.496	0.467	0.524	0.015	9128.879	33.802	0.000	***
SEAS2	-0.141	-0.170	-0.112	0.015	9171.479	-9.615	0.000	***
SEAS3	-0.429	-0.461	-0.396	0.017	9184.220	-25.512	0.000	***
SEAS4	-0.223	-0.258	-0.189	0.017	9192.828	-12.795	0.000	***
DISCL2_CLM	-0.230	-0.373	-0.087	0.073	8091.243	-3.147	0.002	**
DISCL2_CLM_CLM	-0.132	-0.272	0.009	0.071	1912.576	-1.840	0.066	.
DISCLM	-0.576	-0.789	-0.362	0.109	3730.676	-5.289	0.000	***
DISCLX_CL2	0.045	-0.256	0.345	0.154	3052.689	0.290	0.772	
DISCLX_CLM	-0.288	-0.499	-0.077	0.108	4901.572	-2.674	0.008	**
DISnan_CL2	-0.026	-0.368	0.316	0.175	1800.239	-0.149	0.882	
DISO3_CL2	-0.420	-0.632	-0.207	0.109	8071.424	-3.866	0.000	***
DISO3_CLM	-0.590	-0.832	-0.348	0.123	5233.271	-4.780	0.000	***

Table key: ci: 95% confidence interval; SE: standard error; DF: degrees freedom; Sig, significance, with * indicating 95% confidence level, ** indicating 99% confidence level, and *** indicating above the 99.9% confidence level. *Table notes:* Estimates for interaction coefficients not included for brevity.

Table S9 — Prior odds-ratio estimates for risk of DBP-exposure (vs bladder cancer and reproductive anomalies)

Condition	Source	THM4 Exposure (µg/L)	THM4 Baseline (µg/L)	Subset	OR Estimate
**Bladder cancer** (Hrudey et al. 2015)	King & Marrett 1996	>25	<25	-	1.4
	King & Marrett 1996	>75	<25	-	1.7
	Chevrier et al. 2004	>50	<1	male	3.73
	Chevrier et al. 2004	>50	<1	female	1.55
	Villanueva et al. 2007	>49	<8	male	2.53
	Villanueva et al. 2007	>49	<8	female	1.5
	Cantor et al. 2010	>49	<8	-	1.8
**Reproductive anomalies** (Nieuwenhuijsen et al. 2009)	Bove et al. 1995	>80	<20	-	1.57
	Chisholm et al. 2008	>130	<60	-	1.22
	Hwang et al. 2008	>20	<4	-	1.0
	Nieuwenhuijsen et al. 2008	>60	<30	-	1.43
	Hwang et al. 2008	>20	<4	-	1.81
